# RhoA within myofibers controls satellite cell microenvironment to allow hypertrophic growth

**DOI:** 10.1016/j.isci.2021.103616

**Published:** 2021-12-11

**Authors:** Chiara Noviello, Kassandra Kobon, Léa Delivry, Thomas Guilbert, Florian Britto, Francis Julienne, Pascal Maire, Voahangy Randrianarison-Huetz, Athanassia Sotiropoulos

**Affiliations:** 1 Inserm U1016, Institut Cochin, F-75014 Paris, France; 2 CNRS UMR8104, F-75014 Paris, France; 3 Université de Paris, F-75006 Paris, France

**Keywords:** Biological sciences, Cell biology, Stem cells research, Functional aspects of cell biology

## Abstract

Adult skeletal muscle is a plastic tissue that can adapt its size to workload. Here, we show that RhoA within myofibers is needed for overload-induced hypertrophy by controlling satellite cell (SC) fusion to the growing myofibers without affecting protein synthesis. At the molecular level, we demonstrate that RhoA controls in a cell autonomous manner Erk1/2 activation and the expressions of extracellular matrix (ECM) regulators such as *Mmp9/Mmp13/Adam8* and macrophage chemo-attractants such as *Ccl3*/*Cx3cl1.* Their decreased expression in RhoA mutants is associated with ECM and fibrillar collagen disorganization and lower macrophage infiltration. Moreover, matrix metalloproteinases inhibition and macrophage depletion in controls phenocopied the altered growth of RhoA mutants while having no effect in mutants showing that their action is RhoA-dependent. These findings unravel the implication of RhoA within myofibers, in the building of a permissive microenvironment for muscle hypertrophic growth and for SC accretion through ECM remodeling and inflammatory cell recruitment.

## Highlights

• RhoA within myofibers controls SC fusion and muscle hypertrophic growth
• RhoA controls the expression of *Mmp*s and of macrophage chemoattractants (*Ccl3*/*Cx3cl1*)
• RhoA controls ECM remodeling and macrophage recruitment upon hypertrophy
• Mmp inhibition and macrophage depletion phenocopy the blunted growth of RhoA mutant muscles

## Graphical abstract

**Figure:**
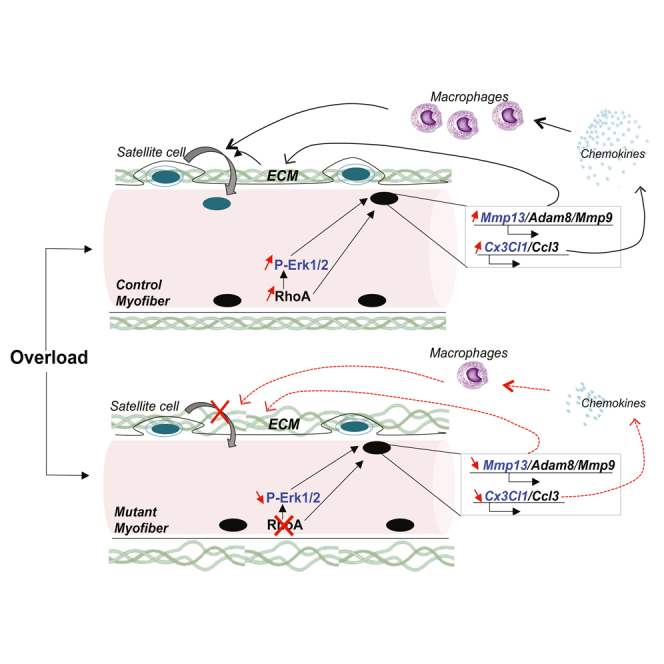

## Introduction

The skeletal muscle is a highly plastic tissue and among the most abundant in the vertebrate body. The cellular muscle unit is the myofiber that is mainly composed of sarcomeric proteins with contractile properties. Physiological demands such as exercise or functional overload (OV) lead to an increase of muscle mass due to a hypertrophic growth of myofibers. In the adult, the two major mechanisms related to muscle growth and sarcoplasmic volume enlargement are: (1) protein synthesis increase to add new contractile filaments to pre-existing sarcomere units, and (2) the fusion of new nuclei provided by resident muscle stem cells, the satellite cells (SCs). In response to increased workload, SCs exit the quiescent state, proliferate, differentiate and subsequently fuse to growing myofibers (Fukada et al., 2020).

Furthermore, at the cellular level, skeletal muscle tissue homeostasis requires the coordinated function of other cell types present in the muscle itself. During the regenerative process, a network of interactions of SCs with different cell types including endothelial cells, fibro/adipogenic progenitors (FAPs), and macrophages orchestrates muscle regeneration (Mashinchian et al., 2018; Wosczyna et al., 2019). Indeed, macrophages play various sequential roles during skeletal muscle regeneration (reviewed in Chazaud, 2020). In the early stages of injury, pro-inflammatory macrophages stimulate SC proliferation and, later on, after their conversion into anti-inflammatory macrophages, they support SC differentiation and fusion into myofibers. However, the contribution of muscle resident cells other than SCs and myofibers to muscle hypertrophy and the role of muscle microenvironment remain poorly studied so far.

In addition, modifications of muscle tissue microenvironment, such as ECM remodeling, occurring upon increased workload have not been fully explored. The major roles of ECM are: (1) to create tissue scaffold for vessels and nerves; (2) to contribute to the force transmission through the passive elastic response of muscle; and (3) to regulate dynamic cell functions through ECM-embedded molecules such as growth factors. In addition, ECM provides a structural support to the integrity of SC niche, physically separating the stem cell pool from other tissue resident cells, and can thus play an important role in SC accretion during hypertrophy. Among the proteins mostly present in ECM are Collagens, Fibronectin (Fn1), and TenascinC (Tnc-C) that are crucial components of SC niche regulating their self-renewal (Bentzinger et al., 2013; Urciuolo et al., 2013) and their regenerative potential (Tierney et al., 2016).

The major enzymes responsible for the physiological breakdown of ECM are matrix metalloproteinases (Mmps). One of the best characterized Mmp in SCs and muscle tissue is Mmp9 (Chenette et al., 2016; Rayagiri et al., 2018). *Mmp9* deletion is deleterious to muscle mass (Mehan et al., 2011) while its constitutive muscle-specific over-expression promotes growth (Dahiya et al., 2011), supporting the notion that a precise regulation of muscle ECM is necessary during periods of adaptation to ensure optimal muscle growth. Nevertheless, how ECM is remodeled and regulated upon increased workload remains unclear.

Ras homolog family member A (RhoA) is a small GTPase protein that oscillates between GTP bound and GDP bound states, regulating a wide spectrum of cellular functions. RhoA controls contractility, actin polymerization, and actin cytoskeleton organization. RhoA is an important player of mechanotransduction that translates physical forces into biochemical signaling pathways and transmits the signal in the nucleus, activating specific transcription factors (Burridge et al., 2019; Lessey et al., 2012). Among them, serum response factor (Srf) activity is regulated by RhoA through the control of actin dynamics (Sotiropoulos at al, 1999). In muscle tissue, mRNA and protein expressions of RhoA have been shown to increase upon chronic functional OV and hypertrophy-stimulating resistance training in rats and humans, respectively (McClung et al., 2003). *In vitro*, cyclic stretch activated RhoA in cultured myotubes (Zhang et al., 2007). However, the functional role of RhoA in skeletal muscle physiology and in muscle mass regulation has not been investigated.

In this study, we assessed the role played by RhoA within myofibers during skeletal muscle hypertrophy by inducing compensatory hypertrophy of *plantaris* muscles harboring a conditional and inducible deletion of *RhoA* in myofibers. We showed compromised hypertrophic growth in the absence of RhoA. The impaired growth was not associated with a protein synthesis defect but rather with altered SC function and fusion to the growing myofibers. We showed that the most down-regulated genes in OV mutant (Mut) muscles compared to controls (Ctl) are those involved in ECM remodeling, like *Mmp9/13* and *Adam8*. Furthermore, a decreased expression of these genes in Mut is associated with un-degraded ECM proteins and fibrillar collagen disorganization. Inhibition of Mmps activity affected muscle growth by preventing SC recruitment and thus phenocopying RhoA mutants. Finally, we showed that the expression of potent chemo-attracting chemokines (*Cx3Cl1* and *Ccl3*) is reduced in *RhoA*-deleted myofibers upon OV and is associated with diminution of macrophage infiltration which functional necessity was demonstrated by depletion experiments.

Altogether our data highlighted a new role of RhoA within myofibers in the regulation of muscle microenvironment upon OV. By modulating ECM remodeling and inflammation, RhoA may allow the constitution of a correct SC niche that will affect SC behavior and thus permits the correct hypertrophic growth.

## Results

### RhoA is required in myofibers for OV-induced hypertrophy

In order to investigate the signaling pathways functionally involved in the control of adult skeletal muscle growth in response to increased load, we performed a transcriptomic study of genes expressed in *plantaris* muscles at the basal state (Sham Operated, SO) and 1 week after OV-induced hypertrophy (OV 1wk). We identified genes differentially expressed before and after OV (fold change >1.2 and pvalue <0.05). Analysis of the canonical pathways using ingenuity pathway analysis (IPA) pointed out RhoA signaling among the first 10 pathways ranked by their significance with predicted activation (*Z* score>2) (Table S1). We thus hypothesized that RhoA signaling could have a crucial role during skeletal muscle hypertrophy.

**Figure 1:**
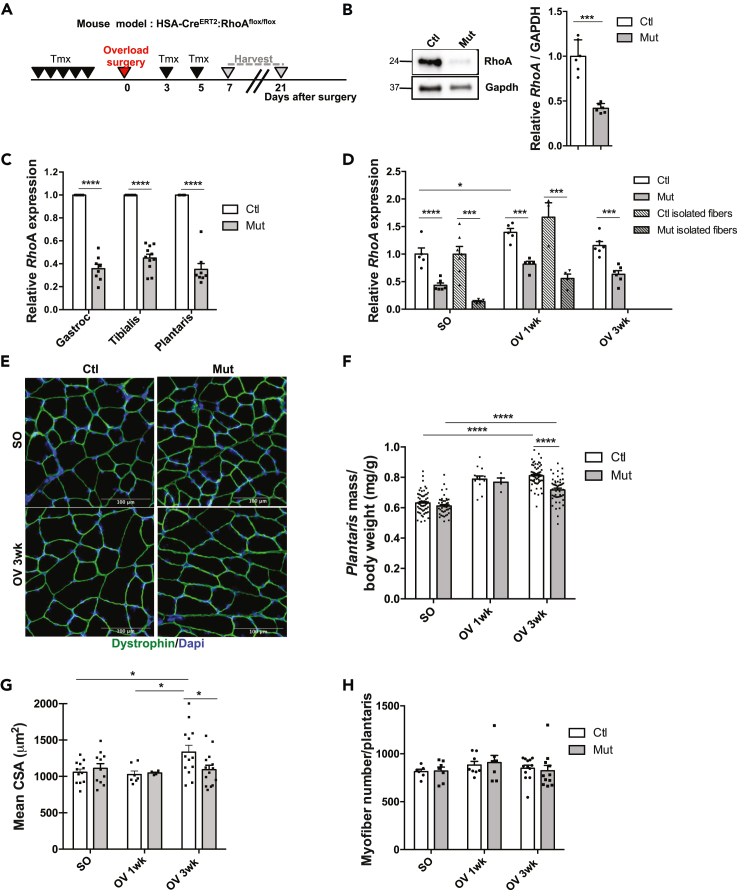
RhoA loss within myofibers impairs overload-induced hypertrophy (A) RhoA mutant (Mut) mice were injected with Tmx 1 week (wk) before OV procedure. *Plantaris* muscles were isolated 1 and 3 wk after surgery. (B) RhoA protein was analyzed by Western blot in Ctl and Mut *plantaris* muscles. Gapdh was used as a loading control. (C) Analysis of *RhoA* mRNA expression by RT-qPCR in Ctl and Mut *gastrocnemius, tibialis anterior* and *plantaris* muscles. Data were normalized by *Hmbs* expression and relative to Ctl (n = 6–11). (D) Analysis of *RhoA* mRNA expression by RT-qPCR in Ctl and Mut *plantaris* muscles or isolated myofibers before (SO) and after 1 and 3wk OV. Data were normalized by *Hmbs* expression and relative to Ctl SO (n = 3–7). (E)*Plantaris* sections immunostained for Dystrophin (green) and nuclear staining with DAPI for Ctl and Mut before (SO) and after 3 wk OV. Scale bar 100 μm. (F) Ratio of *plantaris* mass (mg) to body weight (g) before (SO) and after 1 and 3 wk OV in Ctl and Mut (n = 5–20). (G) Mean CSA (μm^2^) before (SO) and after 1 and 3 wk OV in Ctl and Mut (n = 6–14). (H) Mean myofiber number before (SO) and after 1 and 3 wk OV in Ctl and Mut. Data are mean ± SEM. ∗p value<0.05, ∗∗∗p value<0.001, ∗∗∗∗p value<0.0001. See also Figure S1.

To examine the contribution of RhoA during muscle hypertrophic growth, we generated a mouse model in which *RhoA* is deleted in a specific and inducible manner only in the adult myofiber compartment by injecting tamoxifen (Tmx) to *HSA-Cre*^*ERT2*^*:Rho*^*flox/flox*^ mice (referred to as mutant) and performed OV-induced hypertrophy of the *plantaris* muscle (Figure 1A). At the steady state (SO), a significant 60% RhoA loss was achieved at the protein and transcript levels in different muscles including *gastrocnemius, tibialis anterior*, and *plantaris* (Figures 1B and 1C). Because different cell types are present in the muscles and myofiber myonuclei represent roughly 50% of the total nuclei (Dos Santos et al., 2020), we checked the expression of *RhoA* transcripts in isolated control (Ctl) and mutant (Mut) *plantaris* single myofibers. We showed an efficient 85% mRNA decrease upon Tmx treatment (Figure 1D, SO condition). No difference of muscle weight of myofiber mean cross -section area (CSA) and of myofiber size distribution was observed before hypertrophy between Ctl and Mut *plantaris* (Figures 1E, 1F, 1G, and S1A left panel, SO condition). Following OV, *RhoA* expression increased at the onset of the hypertrophy process (1wk after surgery) in muscle tissue and isolated myofibers (Figure 1D) in agreement with the increased *RhoA* expression observed in overloaded rat muscles (McClung et al., 2003). Three weeks after OV, *plantaris* muscle mass and mean CSA were increased in Ctl as compared to unloaded muscles (SO Ctl). However, the extent of hypertrophic growth was lower in Mut *plantaris* muscles (Figure 1F), mean CSA of overloaded mutant myofibers was significantly smaller compared to the control myofibers (Figure 1G) and, concerning their size, there was a shift toward smaller myofibers upon OV in Mut (Figure S1A right panel, OV condition). Mut *plantaris* muscles displayed a 15% increase in muscle weight (after 3 wk OV) instead of 30% in Ctl and no significant increase of their CSA (Figures 1E, 1F, and 1G). Following hypertrophy, myofiber type distribution was similar between Ctl and Mut suggesting that the smaller CSA of Mut myofibers observed upon OV was not due to changes in myofiber typology (Figure S1B). In addition, the increase in muscle mass was mainly caused by myofiber hypertrophy as the total number of myofibers did not vary after OV (Figure 1H). These data show that RhoA in the myofibers is necessary for optimal myofiber OV-induced hypertrophy and that its absence causes muscle growth defect.

### RhoA is dispensable for protein synthesis control upon hypertrophy

Hypertrophic growth is associated with an increase of protein synthesis and a decrease of protein degradation at the onset of compensatory hypertrophy procedure. Akt signaling plays a central role in this control by activating mTOR pathway and reducing FoxO-mediated atrogenes transcription (Sandri et al., 2004). So, we first tested whether the impaired OV-induced hypertrophy of Mut *plantaris* muscles could be attributed to impaired global protein synthesis. The rate of total protein synthesis was measured *in vivo* using the SUrface SEnsing of Translation (SUnSET) technique that relies on the incorporation of puromycin in newly translated proteins. By Western blot, we showed a similar augmentation of proteins that incorporated puromycin (normalized by the protein total amount) in both Ctl and Mut muscles 1wk following OV, which came back to SO levels after 3wk, with no significant difference between the two groups (Figure S2A). We then analyzed Akt and p70S6K signaling and we observed no difference in Akt and p70S6K phosphorylations between Ctl and Mut, with a 6-fold and 2.5-fold increase upon OV respectively (Figure S2B). These findings suggest that the signaling pathways leading to the activation of protein synthesis are not affected by *RhoA* deletion. However, these experimental approaches do not permit to monitor the absolute level of translation per myofiber that depends on the signaling activating translation and on other parameters such as the amount of mRNA available. To assess this parameter, we performed, on muscle sections, immunostaining for phosphorylated S6 protein that is used as an indicator of PI3k/Akt/mTOR pathway and indirectly protein synthesis. The quantification of pS6 signal intensity (at 1wk after OV) per myofiber showed a trend toward a decrease in the Mut as compared to the Ctl (Figure S2D). We cannot exclude that this tendency to a decreased absolute protein synthesis may participate to the altered hypertrophic growth of RhoA mutants.

By phosphorylating FoxO transcription factors, Akt activation prevents their nuclear localization and the transcription of their target genes, among which are the atrogenes encoding MuRF1 and MAFbx ubiquitin ligases. In line with the activation of Akt, *MuRF1* and *MAFbx* expressions decreased in a similar manner in Ctl and Mut *plantaris* 1wk after OV and came back to basal levels after 3wk (Figure S2C). Altogether, these data suggest that the deletion of RhoA in the myofibers does not affect protein synthesis and degradation pathways upon OV-induced hypertrophy.

### Loss of RhoA in myofibers impairs SC behaviors and in particular their fusion

**Figure 2:**
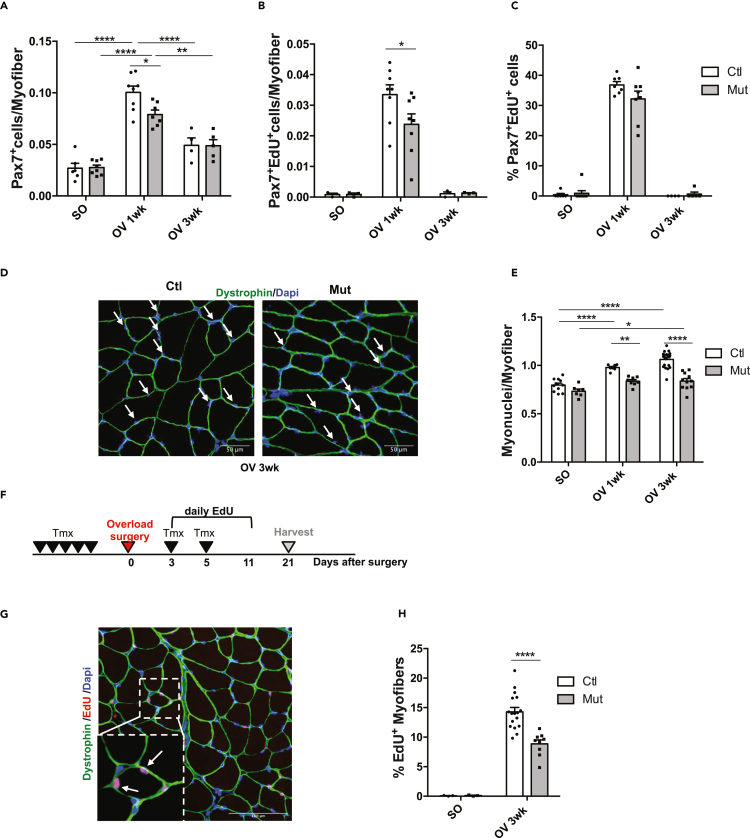
RhoA loss within myofibers impairs SC fusion (A)Number of Pax7^+^ cells per myofiber (n = 4–8) (A). (B) Number of Pax7+EdU + cells per myofiber (n = 3–8). (C) Percentage of Pax7+EdU + among Pax7+ cells (n = 4–8) in Ctl and Mut plantaris sections before (SO) and after 1 and 3wk OV. (D) Plantaris sections immunostained for Dystrophin (green) and nuclear staining with DAPI for Ctl and Mut after 3wk of OV. White arrows indicate myonuclei within Dystrophin + sarcolemma. Scale bar 50 μm. (E) Number of myonuclei within sarcolemma per myofiber before (SO) and after 1 and 3wk OV (n = 7–19). (F) Ctl and Mut mice were injected daily with EdU from day 3–11 after OV. Plantaris were isolated after 3 wk OV. (G) Representative image of Plantaris Ctl section immunostained for Dystrophin (green), EdU (red) and nuclear staining with DAPI after 3wk OV. Fusion was scored as an EdU+ nucleus within a Dystrophin+ myofiber as depicted by the arrows. Scale bar 100 μm. (H) Percentage of EdU + myofibers in Ctl and Mut plantaris sections before (SO) and after 3wk OV (n = 9–14). Data are mean ± SEM. ∗p value<0.05, ∗∗p value<0.01, ∗∗∗∗p value<0.0001. See also Figure S3.

In addition to the increase of protein content, muscle compensatory hypertrophy relies on the accretion of new nuclei through the mobilization of SCs. We hypothesized that RhoA in the myofibers may control the behavior of SCs under OV-induced hypertrophic conditions. We investigated whether RhoA loss in myofibers altered the number of SCs, expressing Pax7 and RhoA. Before OV, Pax7^+^ SC number was identical between Ctl and Mut (Figure 2A, SO). One week after OV, there was a significant increase in Pax7^+^ SCs in both genotypes, but the number of SCs was slightly lower in Mut muscles. We further assessed the proliferative potential of SCs by determining the number of SCs that entered S-Phase (*in vivo* EdU injection 24 hr and 4hr prior the end of the experiment – Pax7^+^EdU^+^ cells) and showed that the number of Pax7^+^EdU^+^ proliferating SCs is slightly reduced in the Mut muscles. However, the proportion of proliferating SCs (Pax7^+^EdU^+^/Pax7^+^ cells) was not significantly affected (Figures 2B and 2C). Overall, following an increased load, RhoA in myofibers may slightly affect the proliferative and/or the activation potential of SCs.

We next investigated whether *RhoA* deletion in the myofiber could affect SCs myogenic differentiation potential and their fusion to the Mut myofibers. Upon OV, *Myogenin* transcript levels did not differ between Ctl and Mut *plantaris*, with a sharp increase 1wk after OV that came back to SO levels after 3wk (Figure S3). Thus, the loss of RhoA within myofibers did not affect the engagement of SCs in myogenic differentiation. Then, the fusion potential of SCs to the growing myofibers was assessed by counting the number of myonuclei within the dystrophin-labeled myofiber sarcolemma (Figure 2D). The number of myonuclei per myofiber was similar before OV between Ctl and Mut and significantly increased 1 and 3 wk after OV in Ctl and to a lesser extend in Mut muscles 3 wk after OV (Figure 2E). Importantly, in Mut *plantaris*, the number of myonuclei was significantly decreased as compared to Ctl 1 and 3 wk after OV (Figure 2E).

To further assert the impaired fusion capacities of SCs to the Mut myofibers *in vivo*, we chronically injected mice subjected to compensatory hypertrophy with EdU and we tracked EdU^+^ nuclei incorporated into growing myofibers (Figures 2F and 2G). This experiment was designed to track the newly fused myonuclei that are derived from cycling SC (as myofibers are postmitotic). Only the nuclei inside the dystrophin-stained sarcolemma were considered and counted (Figure 2G). Three weeks after OV, the percentage of myofibers harboring EdU+ myonuclei was significantly reduced in Mut compared to Ctl (Figure 2H). These data suggest that within myofibers RhoA is needed for an efficient mobilization of SCs during hypertrophy by controlling their number and their recruitment to the growing myofibers. The altered behavior of SCs in absence of RhoA could account, at least in part, for the impaired growth observed in mutant.

### RhoA control of SC behaviors is environment dependent

To investigate whether the altered behaviors of SCs, especially cell fusion, in *RhoA*-deleted muscles subjected to OV are cell autonomous and unrelated to the local environment and/or mechanical stimuli, we set up an *in vitro* assay. This assay was designed to assess whether the absence of RhoA within myotubes (Adeno-Cre-mCherry transduced *RhoA*^*flox/flox*^ myotubes) affects their fusion with RhoA-expressing mononucleated myocytes (expressing GFP) (Figures S4A and S4C). We observed that the lack of *RhoA* expression in myotubes (Figure S4B) did not alter the ability of myocytes to fuse to myotubes *in vitro* (dual-labeling) (Figure S4D). These data suggest that the defect on SC recruitment to *RhoA* mutant myofibers observed *in vivo* relies on muscle environment linked to the *in vivo* OV context, conditions that cannot be reproduced *in vitro*. This highlights the importance of RhoA *in vivo* context under mechanical cues and of *in vivo* microenvironment in the control of muscle compensatory growth. To further analyze how RhoA in the myofibers may control hypertrophic growth, we focused on *in vivo* studies.

### Mmps and chemokines expression is affected in absence of RhoA within myofibers

**Figure 3:**
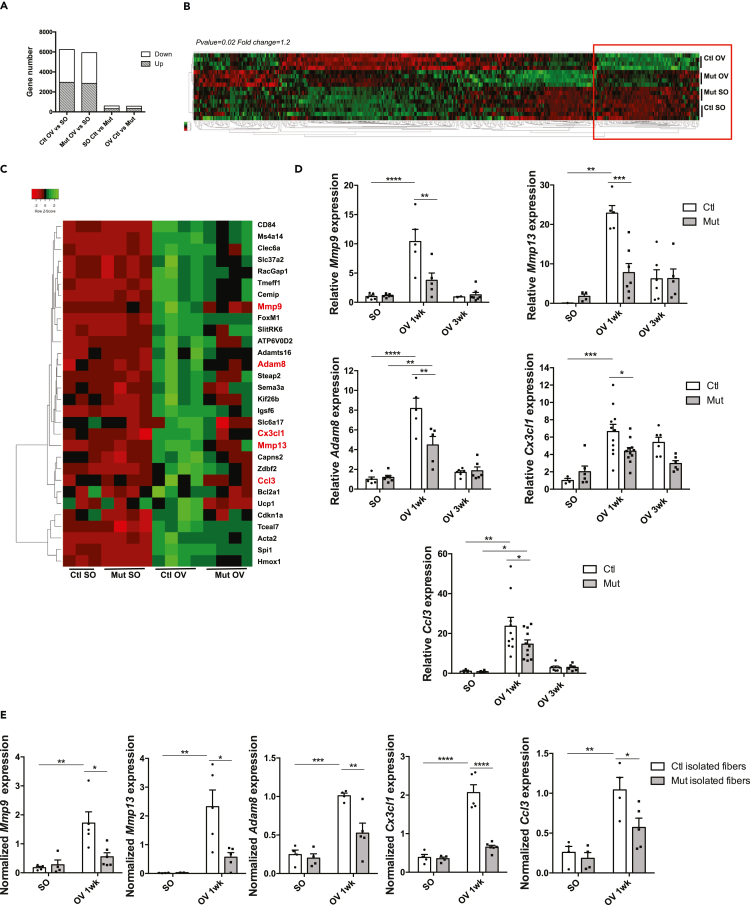
Transcriptomic analysis of control and RhoA mutant overloaded *plantaris* muscles (A) Affymetrix analysis has been performed from RNA extracted from Ctl and Mut *plantaris* before (SO) and after 1 wk OV. Number of genes differentially expressed (fold change>1.2; p value<0.05) depending on the condition SO/OV and on the RhoA expression Ctl/Mut. (B) Heatmap representing up- and down-regulated genes between Ctl and Mut *plantaris* before and after 1wk OV. Genes whose expressions increased upon OV in Ctl are highlighted with a red box. (C) Close-up on the top 30 differentially expressed genes between Ctl OV and Mut OV. (D) Analysis of *Mmp9*, *Mmp13*, *Adam8*, *Ccl3* and *Cx3Cl1* mRNA expression by RT-qPCR in Ctl and Mut *plantaris* before (SO) and after 1 and 3wk OV (n = 3–10). Data were normalized by *Hmbs* expression. (E) Analysis of *Mmp9*, *Mmp13*, *Adam8*, *Ccl3*, and *Cx3Cl1* mRNA expression by RT-qPCR in Ctl and Mut isolated myofibers from *plantaris* muscles before (SO) and after 1 week OV (n = 4–6). Data were normalized by *Hmbs* expression. Data are mean ± SEM. ∗p value<0.05, ∗∗p value<0.01, ∗∗∗p value<0.001, ∗∗∗∗p value<0.0001. See also Table S4.

In order to decipher the molecular mechanisms underlying the growth defects of *RhoA*-deleted myofibers, we performed a genome-wide microarray analysis of gene expression from Ctl and Mut *plantaris* muscles, before and 1wk after OV procedure. Gene expression profiling evidenced that a massive change occurred in muscles upon increased load, with more than 6000 genes up- and down-regulated in Ctl and in Mut muscles (SO vs OV 1wk; fold change>1.2; pvalue<0.05). Upon OV, 569 genes were differentially expressed between Ctl and Mut *plantaris* (Figure 3A). Among the 233 down-regulated genes in OV Mut muscles as compared to Ctl, we focused our attention on genes up-regulated in Ctl after hypertrophy (and to a lesser extend in mutants) (Figure 3B, highlighted by a red box). The 30 most differentially expressed genes of this category (Mut OV vs Ctl OV) are shown in Figure 3C. Among them, we identified genes encoding proteins involved in ECM remodeling, such as *Mmp9* gelatinase, *Mmp13* collagenase, and *Adam8* metalloproteinase, and important chemokines such as *Cx3cl1* and *Ccl3* (Figure 3C). We confirmed by RT-qPCR that these genes were strongly and transiently expressed upon OV in Ctl while their expression was significantly reduced in Mut (Figure 3D).

To determine whether the altered expression of these genes could be linked to the absence of RhoA in myofibers or to other cell types expressing RhoA present in the muscle, we isolated single myofibers from Ctl and Mut *plantaris* before and following OV. In line with the data on bulk RNA, we showed that expression levels of *Mmp9, Mmp13, Adam8, Cx3Cl1*, and *Ccl3* genes were strongly increased in Ctl single myofibers following OV and were significantly diminished in Mut (Figure 3E).

**Figure 4:**
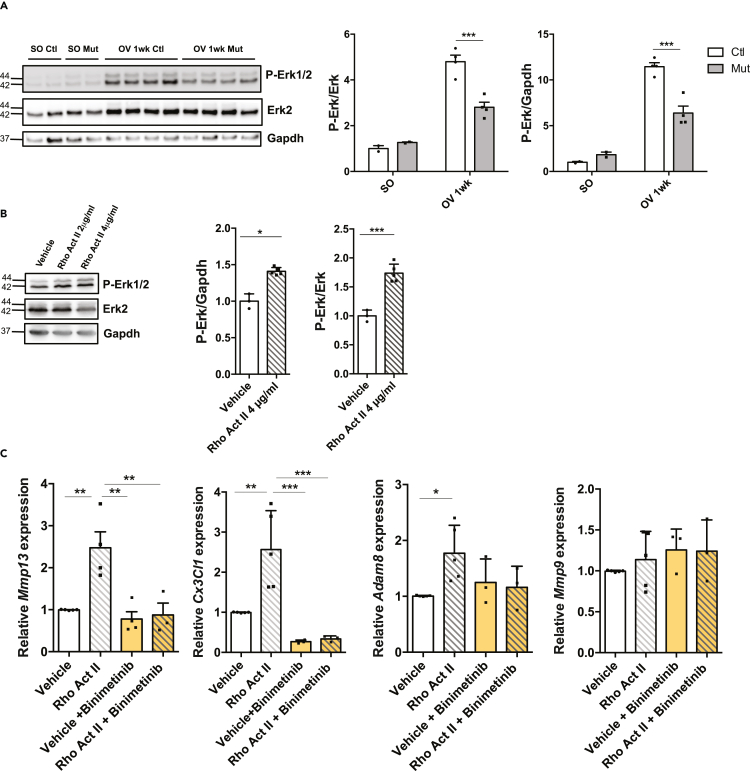
RhoA loss within myofibers impairs Erk1/2 activation upon overload-induced hypertrophy (A) Phosphorylated Erk1/2 and total Erk2 were analyzed by Western blot in Ctl and Mut *plantaris* before (SO) and after 1wk OV (n = 4). Gapdh was used as a loading control. Ratio of the quantification of P-Erk1/2 to Erk1/2 relative to Ctl SO and ratio of the quantification of P-Erk1/2 to Gapdh are shown in the right panels. (B) Phosphorylated Erk1/2 and total Erk2 were analyzed by Western blot in Ctl myotubes treated with Rho Activator II or vehicle for 3 hr (n = 3–5). Gapdh was used as a loading control. Ratio of the quantification of P-Erk1/2 to Erk1/2 and ratio of the quantification of P-Erk1/2 to Gapdh are shown in the right panels. (C) Analysis of *Mmp9*, *Mmp13, Adam8*, and *Cx3Cl1* mRNA expression by RT-qPCR in Ctl myotubes treated with Rho Activator II (4 μg/mL) and/or Erk inhibitor (Binimetenib) for 8hr (n = 3–4). Data were normalized by *Hmbs* expression. Data are mean ± SEM. ∗p value<0.05, ∗∗p value <0.01, ∗∗∗ p value<0.001.

Moreover, we performed a transcriptomic analysis of gene differentially expressed between *RhoA*^*flox/flox*^ myotubes expressing (Ad-mCherry transduced) or not RhoA (Ad-Cre-mCherry transduced). There was very little overlap between the genes differentially expressed in myotubes in culture expressing or not RhoA and the genes differentially expressed in Ctl and Mut OV *plantaris* muscles (Mut OV 1wk vs Ctl OV 1wk) (41 genes) or Ctl and Mut SO muscles (43 genes). The expression of only 25 and 34 genes varied in the same direction (up or down) in the common genes with OV and SO conditions respectively (Figure S4E, Tables S2 and S3). *Mmp9, Mmp13, Adam8*, and *Cx3Cl1* genes were not among them, indicating that their expression was not affected by *RhoA* deletion in basal culture conditions (Figure S4B, Tables S2 and S3). Together, these findings further bring out that the deletion of RhoA in myotubes *in vitro* cannot recapitulate the *in vivo* setting both at basal state and following hypertrophy. One parameter that is lacking *in vitro,* as compared to *in vivo*, is the mechanical load applied to the cultured myotubes that could be associated with low RhoA activity. Indeed it has been shown that stretch can activate RhoA in myotubes (Zhang et al., 2007). To activate RhoA in standard culture conditions, we treated myotubes with a RhoA activator (Rho Activator II) and we monitored gene expression after 8 hr. Importantly, the expression levels of *Mmp13, Adam8,* and *Cx3Cl1* were significantly increased following RhoA activation (Figure 4C). These observations are in line with the data obtained in isolated single myofibers (Figure 3E). They suggested that at least a part of the impaired expression of these genes *in vivo* can be attributed to the specific absence of RhoA in myofibers and is thus cell autonomous. We hypothesized that the decreased expression of those genes in myofibers upon OV might participate in the incorrect hypertrophic response of mutant muscles.

### Pathways impaired by RhoA depletion in overloaded myofibers

To obtain further insights on the pathways that might be impaired by *RhoA* deletion, we focused our attention on the upstream regulators predicted activated or inhibited following OV in Ctl, but not in Mut muscles using IPA analysis (*Z* score>2 or <2, and pvalue<0.05). Several upstream regulators were involved in the remodeling of ECM such as Mmp9 and Tnc-C (Table S4). Mmp9 stood as an interesting molecule because: (1) it is one of the genes whose expression was the most decreased in Mut OV as compared to Ctl OV (Figure 3C), (2) it is among the Upstream Regulator predicted activated only in the Ctl, and (3) it is connected to the predicted activation/inhibition of other several Upstream Regulators such as Mmps substrates Tnc-C, Fgf, Tnf, Cxcl1 and SerpinA4 (Table S4). Mmps are proteolytic enzymes that hydrolyze components of the ECM participating to its remodeling and release many bioactive cytokines and growth factors entrapped in ECM (Page-McCaw et al., 2007). For instance, the release and activation of Tnfα and the matrix-associated growth factor Fgf by Mmp9 have been reported and consistently their activities were predicted to be activated in our IPA analysis (Table S4) (Allen et al., 2003). In the case of SerpinA4, its cleavage by Mmp9 inactivates Serpin and accordingly its activity was predicted to be decreased (*Z* score = −2.17) (Table S4) (Liu et al., 2000).

In addition, several upstream regulators, predicted activated only in Ctl but not in Mut after OV, were associated with inflammation such as IL3, IL17RA and Gm-csf (implicated in the functional activation and survival of macrophages) and chemokines Ccl2, Cxcl1, chemokine receptors CcR1 (Ccl3 receptor) and CcR2 (Ccl2 receptor) participating to macrophage recruitment (Table S4).

Finally, several signaling pathways including Erk1 (Mapk3), Jnk (Mapk8), Stat3, Nfkb (RelA and Sn50 peptide) were predicted activated in Ctl OV *plantaris* and not in Mut OV (Table S4). We thus checked by Western blot their activity (Erk1/2, Stat3, JNK) in OV Ctl and OV Mut muscles. A difference between Ctl and Mut was only observed for Erk1/2 activity. Indeed, the increase of P-Erk1/2 following OV is significantly less in Mut as compared to Ctl muscles (Figure 4A). In order to investigate whether RhoA can activate directly Erk1/2 within myofibers, we treated Ctl myotubes *in vitro* with a Rho activator. Interestingly, a short activation of Rho GTPases (3hr) was sufficient to induce an increase of P-Erk1/2 in a dose-dependent manner (Figure 4B) suggesting that the decrease of P-Erk1/2 observed in the OV Mut muscles could be partially attributed to the lack of RhoA in myofibers. Interestingly, the inhibition of Erk signaling using Binimetinib blunted the increase of *Cxcl3* and *Mmp13* expression induced by RhoA activation in Ctl myotubes (Figure 4C). In contrast to what was observed *in vivo*, no change in *Mmp9*mRNA expression was observed *in vitro* following RhoA activation (Figure 4C) suggesting that *Mmp9* expression may be dependent on *in vivo* microenvironment and that *in vitro* RhoA activation “alone” may not be sufficient.

Altogether, these data suggest that upon hypertrophy, RhoA in the myofibers might affect pathways involving Erk1/2 signaling, ECM remodeling and macrophage recruitment/activity.

### RhoA in myofiber impairs ECM organization and Mmps activity

**Figure 5:**
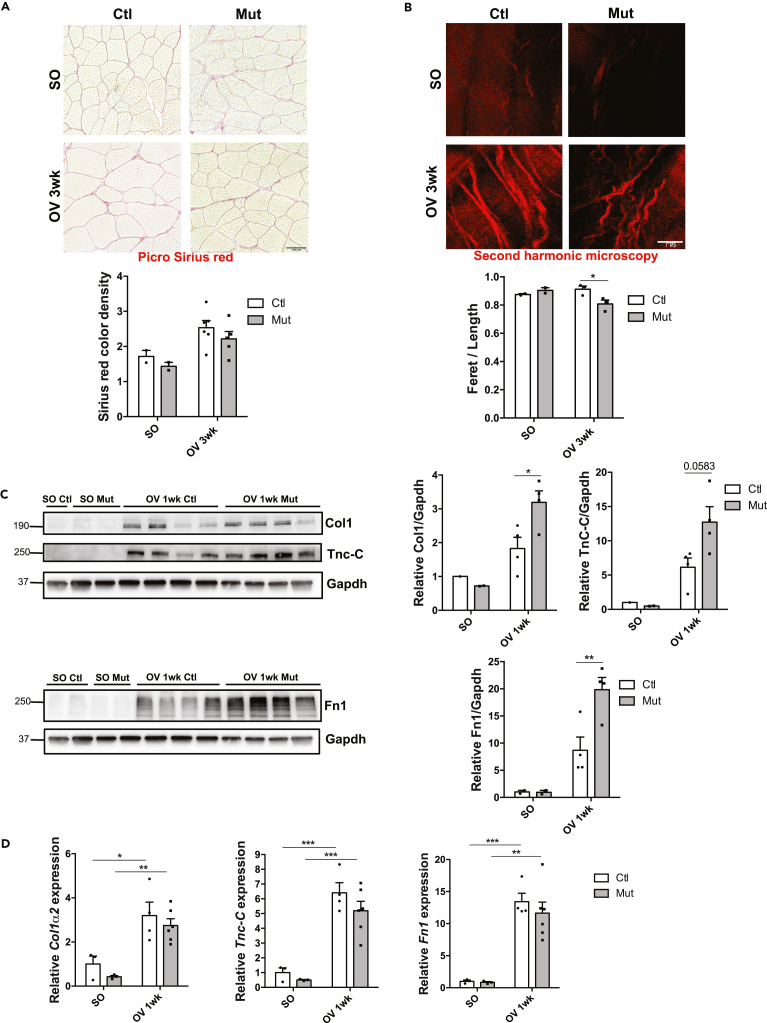
RhoA loss within myofibers impairs ECM organization upon overload-induced hypertrophy (A) Picrosirius red staining of *plantaris* muscle sections from Ctl and Mut mice before (SO) and after 3wk OV. Quantification of color density was performed using CaseViewer and shown in the lower panel (n = 2–5). Scale bar 100 μm. (B) Visualization of fibrillar collagen using second harmonic generation imaging on thick *plantaris* muscle sections from Ctl and Mut before (SO) and after 3 wk OV. Tortuosity quantification (ratio Feret to length of one collagen fiber) from Ctl and Mut before (SO) and after 3 wk OV (n = 3) is shown in the bottom panel. Scale bar 5 μm. (C) Collagen1, Tenascin-C and Fibronectin were analyzed by Western blot in Ctl and Mut *plantaris* before (SO) and after 1 wk OV. Gapdh was used as a loading control (n = 3–4). Ratio of the quantification of Col1, TnC and Fn1 to Gapdh and relative to Ctl SO is shown in the right panel. (D) Analysis of *Col1α2*, *Tnc*, and *Fn1* mRNA expression by RT-qPCR in Ctl and Mut *plantaris* before (SO) and after 1 and 3 wk OV (n = 3–4). Data were normalized by *Hmbs* expression. Data are mean ± SEM. ∗p value<0.05, ∗∗p value<0.01, ∗∗∗p value<0.001, ∗∗∗∗p value<0.0001.

Based on the transcriptomic and IPA analyses, we hypothesized that ECM remodeling and Mmp activity might be affected by RhoA loss in the growing myofibers. We demonstrated that the global collagen content (quantified by Picrosirius red staining) increased in a similar extent upon OV in both Ctl and Mut muscles (Figure 5A). To further analyze ECM organization, we investigated ECM biophysical aspect by performing second harmonic generation (SHG) imaging that allows the visualization of fibrillar collagen (Figure 5B). We observed that fibrillar collagen appears more tortuous in Mut compared to Ctl 3wk after OV, as shown by the decrease of the ratio of fibrillar collagen Feret’s diameter to length (Figure 5B lower panel). Moreover, it accumulated between the myofibers rather than surrounding them. These data suggest that in hypertrophic conditions RhoA impacts ECM organization in terms of tortuosity and assembly.

Because Mmps are key proteolytic enzymes that hydrolyze components of the ECM, we next quantified the protein amount of ECM substrates of Mmp9 and Mmp13 such as Collagen1 (Col1, Tnc-C and Fn1 by Western blot (Figure 5C)). We observed that Col1, Tnc-C and Fn1 protein amounts augmented with OV in both Ctl and Mut muscles and that there was a further significant accumulation of these proteins in Mut OV muscles. Interestingly, the sharp increase upon OV in both genotypes was also seen at mRNA level indicating that this may mainly be due to increased transcription of these genes (Figure 5D). Importantly, there was no difference in transcript level between OV Ctl and OV Mut *plantaris* suggesting that the increase in Col1, Tnc-C and Fn1 proteins amounts observed in the Mut might be due to their decreased cleavage by Mmp9 and Mmp13 (Figure 5D).

Thus, we could speculate that in the absence of RhoA, Mmp expression diminution might affect the correct ECM protein hydrolysis and that a wrong ECM rearrangement could impair muscle growth.

### Mmps are functionally involved in compensatory hypertrophy

**Figure 6:**
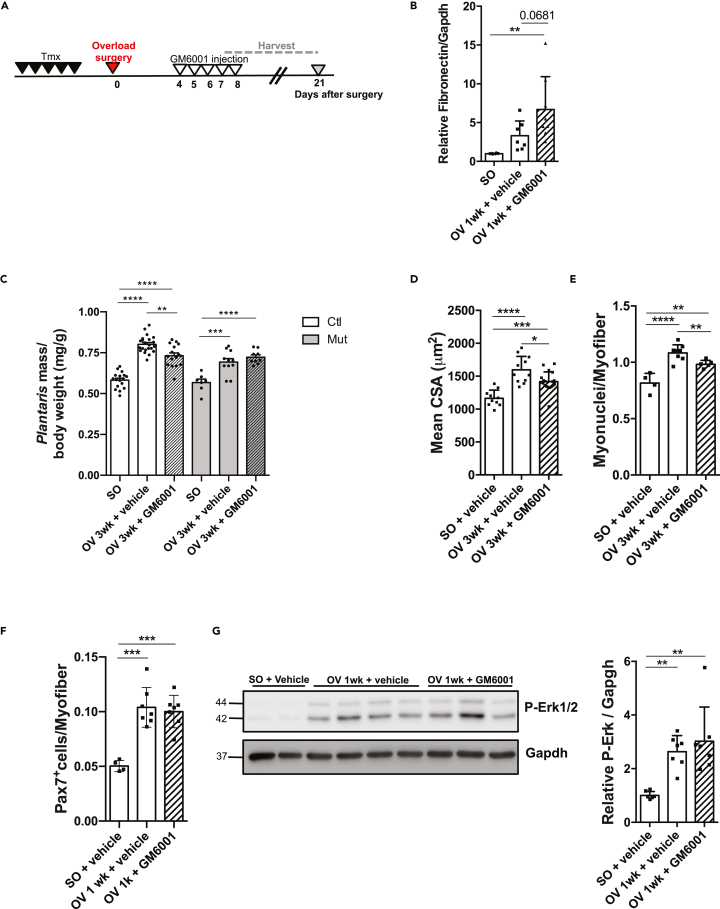
Mmps activity are important for overload-induced hypertrophy (A) Ctl and Mut mice were submitted to OV and injected with Mmp inhibitor (GM6001) or vehicle from day 4–8 after surgery. *Plantaris* muscles were isolated after 1 and 3 wk OV. (B) Fibronectin was analyzed by Western blot in Ctl and Mut *plantaris* before (SO) and after 1wk OV. Gapdh was used as a loading control. Ratio of the quantification of Fn1 to Gapdh relative to Ctl SO is shown (n = 6–8). (C) Ratio of *plantaris* mass (mg) to body weight (g) before (SO) and after 3 wk OV in Ctl and Mut treated or not with GM6001 (n = 7–14). (D) Mean CSA (μm^2^) before (SO) and after 3 wk OV in Ctl treated or not with GM6001 (n = 4–14). (E) Number of myonuclei within sarcolemma per myofiber in *plantaris* sections before (SO) and after 3 wk OV from Ctl treated or not with GM6001 (n = 4–8). (F) Number of Pax7^+^ cells per myofiber in *plantaris* sections before (SO) and after 1 week OV from Ctl treated with GM6001 or vehicle (n = 4–7). (G) Phosphorylated Erk1/2 was analyzed by Western blot in Ctl treated or not with GM6001 before and 1 wk OV. Gapdh was used as a loading control. Ratio of the quantification of P-Erk1/2 to Gapdh is shown in the right panel (n = 3–8). Data are mean ± SEM. ∗p value<0.05, ∗∗p value<0.01, ∗∗∗∗p value<0.0001.

To get insights on the functional implication of Mmps in the hypertrophic process, we treated Ctl and Mut mice using the broad spectrum Mmp inhibitor GM6001 (targeting Mmp1, 2, 9, 8, 3) and we assessed its effect on compensatory hypertrophy (Figure 6A). One week after OV, Fn1 protein amount, a common substrate of several Mmps, significantly increased only in GM6001-treated muscles, showing the *in vivo* efficacy of the drug (Figure 6B). In addition, to further verify *in vivo* the efficacy of the drug, we measured Mmps gelatinase activity upon OV in muscle sections before and after GM6001 injection using *in situ* zymography (Figure S5) (Yeghiazaryan et al., 2012). As expected, there was an increase of interstitial and nuclear Mmp activity staining in OV muscles as compared to SO. In addition, after GM6001 treatment the intensity of interstitial staining in overloaded muscles decreased (Figure S5) showing the efficacy of the drug. Importantly, *plantaris* muscles of GM6001-treated Ctl mice were less hypertrophied 3wk after OV than muscles of vehicle-injected mice as assessed by their decreased weight, while GM6001 treatment had no effect on RhoA mutant overloaded muscle weights as compared to vehicle-treated muscles (Figure 6C). The lack of effect of GM6001 on Mut muscle hypertrophy supports the fact that Mmps operate downstream RhoA.

In the case of Ctl *plantaris*, their altered hypertrophic growth following GM6001 treatment was accompanied by a reduction of CSA and of the number of myonuclei per myofiber (Figures 6D and 6E). To clarify whether the reduced fusion was due to SC reduced number, we quantified the number of Pax7+ cells in GM6001-treated Ctl mice and observed no difference with vehicle-treated mice (Figure 6F), suggesting that the inhibition of Mmps from the fourth day of OV affected mainly SC fusion and not their number.

Altogether these results show the important role of Mmps to achieve optimal hypertrophy in response to increased load. As Mmp inhibition in Ctl phenocopied the altered growth and SC fusion observed in RhoA mutant muscles, as Mmp inhibition did not further affect Mut impaired hypertrophic growth and as *Mmp9/13* expression was decreased in RhoA mutants upon OV, our findings suggest that the blunted hypertrophy of *RhoA* deleted muscles may be partially due to a decreased Mmp activity.

Mmp processing can release ECM-tethered growth factors and bioactive cytokines that can indirectly activate different signaling pathways. Therefore, we wondered whether the attenuated activation of Erk1/2 observed in RhoA mutant OV muscles (Figure 4A) could be partially attributed to the diminished Mmp expression/activity. Importantly, GM6001-and vehicle-treated muscles displayed similar levels of P-Erk1/2 upon OV suggesting that the alteration of Erk signaling is not downstream of the Mmps (Figure 6G).

### RhoA in overloaded myofibers impacts macrophage recruitment implicated in muscle growth

**Figure 7:**
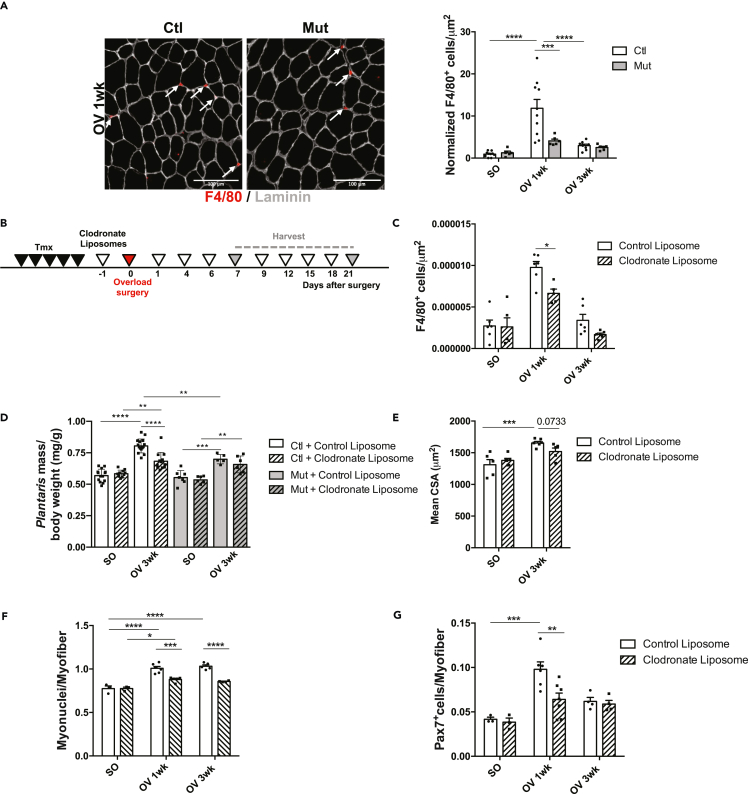
RhoA loss within myofiber impairs macrophage recruitment (A) *Plantaris* sections were immunostained for F4/80 (red), Laminin (gray) and nuclear staining with DAPI for Ctl and Mut after 1 wk OV. White arrows indicated F4/80^+^/DAPI macrophages. Number of macrophages (F4/80^+^) per μm^2^ before (SO) and after 1 and 3wk OV relative to Ctl SO are quantified in the right panel (n = 5–6). Scale bar 100 μm. (B) Ctl and Mut mice were injected with Clodronate-loaded liposomes or control liposomes one day before OV and at day 1, 4, 6, 9, 12, 15, 18 after OV. *Plantaris* muscles were harvested after 1 and 3 wk OV. (C) Number of macrophages (F4/80^+^) per μm^2^ before (SO) and after 1 and 3 wk OV in *plantaris* sections from Ctl treated with Clodronate loaded liposomes or control liposomes (n = 5–6). (D) Ratio of *plantaris* mass (mg) to body weight (g) before (SO) and after 3 wk OV in Ctl and Mut mice treated with Clodronate loaded liposomes or control liposomes (n = 6–10). (E) Mean CSA (μm^2^) before (SO) and after 3 wk OV in Ctl treated Clodronate loaded liposomes or control liposomes (n = 5–6). (F) Number of Pax7^+^ cells per myofiber in *plantaris* sections before (SO) and after 1 and 3 wk OV from Ctl treated with Clodronate loaded liposomes or control liposomes (n = 3–7). (G) Number of myonuclei within sarcolemma per myofiber in *plantaris* sections before (SO) and after 1 and 3 wk OV from Ctl treated with Clodronate loaded liposomes or control liposomes (n = 4–8). Data are mean ± SEM. ∗p value<0.05, ∗∗p value<0.01, ∗∗p value<0.01, ∗∗∗p value<0.001, ∗∗∗∗p value<0.0001. See also Figure S5.

In absence of RhoA, we showed a decreased expression of *Ccl3* and *Cx3cl1* by the growing myofibers (Figures 3D and 3E). These two chemokines/chemo-attractants are implicated in inflammation by recruiting and activating macrophages (Zhao et al., 2016; Maurer and von Stebut, 2004). In addition, IPA analysis highlighted several Upstream Regulators activated in OV Ctl linked to the inflammatory response (Table S4). Therefore, we investigated the number of macrophages (F4/80^+^) present in muscle sections following compensatory hypertrophy. One week after OV, there was a 10-fold increase in macrophage number in Ctl muscles that came back almost to SO levels 3wk after OV, showing a transient and strong inflammatory response in the muscle following increased workload (Figure 7A). Strikingly, F4/80^+^ macrophage number was significantly reduced in Mut compared to Ctl 1wk after OV (Figure 7A). This prompted us to hypothesize that RhoA in the myofibers may influence macrophage recruitment (by controlling indirectly *Ccl3* and *Cx3cl1* expression levels) in the muscle tissue following mechanical load increase.

It has been shown that macrophages are very important to ensure the proper regeneration of muscle by controlling SC behaviors such as proliferation and fusion (Chazaud, 2020). To investigate whether macrophages play a functional role during hypertrophy, we depleted the macrophages by intraperitoneally injecting Clodronate liposomes that induce macrophage death in Ctl and RhoA Mut mice (Figure 7B). First, we checked macrophage depletion efficacy in Ctl mice by counting the number of F4/80^+^ macrophages on *plantaris* sections. We observed a 30% and a 50% decrease 1wk and 3wk after OV, respectively (Figure 7C). This partial depletion was sufficient to blunt hypertrophic growth of Ctl *plantaris* muscles 3wk after OV as evidenced by the reduced *plantaris* weight while having no effect on mutants, suggesting that macrophage recruitment upon OV depends on RhoA activity (Figure 7D). We focus our further analyses on Clodronate-treated Ctl mice and we observed that macrophage depletion led to diminished number and fusion of SCs (Figures 7E, 7F, and 7G), suggesting that, in hypertrophic conditions, macrophages modulate SC behavior. These results are reminiscent of those observed in RhoA Mut overloaded muscles, in which SC behaviors are altered (Figure 2). Altogether these data are in agreement with a putative role of RhoA within the myofiber in the control of muscle growth, by favoring the recruitment of macrophages in overloaded muscles.

## Discussion

Taking advantage of a genetic model that allows the deletion of *RhoA* in myofibers and not in SCs, we demonstrated that RhoA is needed for skeletal muscle hypertrophy and for the recruitment of SCs to the growing fibers. We provided evidence for the implication of RhoA within myofibers in the building of a permissive microenvironment for muscle growth and for SC accretion through ECM remodeling and macrophage recruitment. At the molecular level, we propose that in response to increased workload, RhoA controls in a cell autonomous manner Erk1/2 activation and the expression of ECM regulators such as *Mmp9/Mmp13/Adam8* and of macrophage chemo-attractants such as *Ccl3*/*Cx3cl1* (Figure S6).

Hypertrophic growth of skeletal muscle of young mice relies on both the increase of net protein content and the addition of new nuclei in the growing myofibers provided by SCs as both blunting of protein synthesis signaling by rapamycin treatment (Bodine et al., 2001) and of SC fusion (Goh and Millay, 2017; Guerci et al., 2012; Randrianarison-Huetz et al., 2018), impair myofiber growth upon increased load. We showed that in the absence of RhoA, the hypertrophy defect is not accompanied by a major deregulation of protein synthesis/degradation balance nor by a compromised Akt signaling, but mostly by a deficiency of SC behavior and mainly fusion. We propose that upon OV-induced hypertrophy, the altered recruitment of SC to *RhoA* mutant myofibers may be responsible for their impaired growth rather than their slight decrease of SC number (21%). Interestingly, we have previously shown, in a model of a conditional deletion of Srf in myofibers, that the reduced number of SCs observed (35% reduction) was not the limiting step responsible for the lack of hypertrophy of Srf mutant muscles (Guerci et al., 2012). Indeed, the restoration of SC proliferation did not improve SC fusion and was unable to drive muscle hypertrophy. In contrast, the restoration of SC fusion was sufficient to rescue OV-induced growth of Srf mutant muscles without any increase in SC proliferation. These data support the idea that SC fusion is one of the limiting cellular events to achieve a correct muscle hypertrophy.

The contribution of SCs in skeletal muscle hypertrophic growth has been highly debated and one factor that could explain the discrepancies toward the necessity of new myonuclei accretion is the age of the mice, as young mice (less than 4 months old) and not mature mice (more that 4 months old) require SC fusion to support myofiber hypertrophy (Murach et al., 2017, 2021a). Because age dependence was not the main focus of this study, we pointed our attention on 2- to 3-month-old mice. Moreover, the necessity of competent fusing SCs to have the correct muscle hypertrophy in young mice has been clearly demonstrated using genetic mouse models in which SCs are unable to fuse (Prasad and Millay, 2021). For instance, SC deletion of *Myomaker*, a muscle-specific membrane protein necessary for fusion of muscle progenitors, compromised muscle hypertrophic response (Goh and Millay, 2017). In addition, loss of Srf in SCs, a master regulator of F-actin scaffold, impaired OV-induced muscle growth by the absence of SC fusion and restoration of Srf mutant SC fusion capacities was sufficient to rescue muscle hypertrophic growth (Randrianarison-Huetz et al., 2018).

As previously mentioned, in RhoA mutant muscles, global protein neosynthesis (in whole muscle) and Akt/S6K activities were not significantly affected. Accordingly, when *Srf* was deleted in myofibers, Akt signaling (controlling protein translation) increased in a similar manner in control and mutant upon OV, suggesting that the physiological level of Akt activation upon OV was not as such sufficient to support growth independently of SC accretion (Guerci et al., 2012). Interestingly, when Akt was constitutively activated in mutant myofibers through MyrAkt electroporation, myofiber growth was achieved in absence of increased load and of SC accretion (Guerci et al., 2012). Thus, constant supra-physiological level of Akt activity is sufficient to induce growth in Srf mutants and in absence of SC fusion. According to these findings, we favor the following scenario: Upon increased load, the physiological activation levels of Akt and protein synthesis participate in growth, but in the absence of myonuclei accretion they are not sufficient to support growth unless supra-physiologic activation levels of translational activity are reached.

RhoA is an important small GTPase protein regulating contractility, actin cytoskeleton rearrangement, and actin polymerization through its downstream effector Rho-associated coiled-coil protein kinase and mammalian diaphanous protein among many others. Moreover, RhoA activation responds to mechanical stimuli and mechanical force transmitted and mediated by cell-matrix and cell-cell-interaction (Lessey et al., 2012). In cultured C2C12 myotubes, it has been shown that cyclic mechanical stretch increased RhoA activity (Zhang et al., 2007) suggesting that RhoA participates in the mechanical stretch response in myotubes. To identify the molecular mechanisms underlying the lack of hypertrophic growth of *RhoA* mutant myofibers, we compared the data of microarray experiments performed *in vivo* on SO or OV *plantaris* Ctl and Mut muscles and the data of microarrays performed on differentiated myotubes expressing or not RhoA. The intersection sets were very small, suggesting that the pathways regulated by RhoA *in vivo* (before or during OV) are very different from those operating in myotubes cultured in standard static conditions. The following genes *Mmp9, Mmp13, Adam8, Ccl3, Cx3Cl1,* which were differentially expressed between Clt and Mut overloaded whole *plantaris* muscles and isolated myofibers, were not expressed differentially in myotubes expressing or not RhoA. Additionally, we were not able to reproduce the fusion defects of Ctl myocytes with RhoA mutant myotubes *in vitro*. These discrepancies between *in vivo* and *in vitro* settings highlight the need of mechanical inputs to trigger RhoA-dependent muscle hypertrophy. In addition, in response to increased load, RhoA in the myofibers may affect other cell types present in the muscle, but absent *in vitro*, that may participate in muscle growth. Altogether, this emphasizes the importance of *in vivo* models to analyze RhoA contribution to muscle hypertrophy.

While searching for additional players responsible for the impaired growth of RhoA mutant muscles, we have to consider fibrogenic cells, FAPs and/or macrophages implication. Upon hypertrophy, TCF4^+^ interstitial fibrogenic cells have been shown to functionally interact with SC (Fry et al., 2017). In this study, SC depleted muscles were compared with control muscles and it was shown that SCs secrete exosomes containing miR-206 that regulates a regulator of collagen biosynthesis, Rrbp. In SC-depleted overloaded muscles there was an increase of *Rrbp1, Col1α2, Col3α1*, and *Col12α1* expressions as compared to controls that was not observed in RhoA mutant overloaded *plantaris* muscles. Recently, the same group showed that muscle hypertrophy may be supported by a permissive environment through communication between FAPs (*Pdgfrα* expressing cells) and SCs, independently of SC fusion (Murach et al., 2021b). However, in overloaded *RhoA* deleted muscles, *Pdgfrα* gene expression was not significantly different from Ctl muscles 1wk after OV. Altogether, these observations argue against the implication of fibrogenic/FAPs cells in the impaired growth phenotype of RhoA mutant.

It has been shown that Rho GTPases, and in particular RhoA, are potent activators of Srf transcriptional activity (Sotiropoulos et al., 1999; Hill et al., 1995). In addition, we have previously shown that Srf within the myofibers is required for muscle OV-induced hypertrophy by exerting a paracrine control on SC recruitment to the growing fibers through Ptgs2/IL4 (Guerci et al., 2012). We could wonder whether a decrease of Srf activity could be responsible for the impaired hypertrophic growth of *RhoA* deleted muscles. Several data argue against this possibility. First, our *in vivo* and *in vitro* transcriptomic data on muscles upon OV and on myotubes suggested that RhoA governs downstream signaling pathways independently from Srf, as almost none of the *bona fide* Srf target genes are down-regulated when RhoA is absent. For instance, *Ptgs2* (a Srf target gene), which expression increases during OV in Ctl muscles and is blunted in Srf mutants, displays a similar expression in Ctl and RhoA Mut *plantaris* 1wk after OV. In cultured myotubes, *RhoA* deletion did not affect the expression of Srf target genes such as *Egr1*, *Myl9, Cnn2, Ptgs2* and *Acta1* (Guerci et al., 2012; Randrianarison-Huetz et al., 2018). Second, the expression of several “RhoA responsive” genes such as *Mmp13, Adam8*, and *Cx3Cl1* that increased following chemical activation of RhoA *in vitro* on cultured myotubes and that decreased in overloaded RhoA Mut muscles, remained unaffected by constitutive Srf activation in myotubes (induced by Ad-SrfVP16 transduction) (Guerci et al., 2012). Thus, we propose that the impeded muscle growth of RhoA mutant is likely not due to a decreased Srf activity and that Srf-independent signaling pathways are operating downstream RhoA upon hypertrophy.

In the present study, we showed that genes involved in the ECM remodeling, like *Mmp9* and *Mmp13* are among the genes mostly down-regulated in overloaded muscles in the absence of RhoA. In addition, *in vivo* Mmp inhibition (from day 4 to day 8 after OV) by a broad-spectrum Mmp inhibitor impaired OV-induced hypertrophy and SC accretion without affecting SC number. However, Mmp inhibitor GM6001 was shown to alter SC number following muscle laceration-induced injury (Bellayr et al., 2013). This discrepancy could be attributed to the different muscle homeostasis perturbation (OV versus muscle laceration injury) and timing at which muscles were treated (5 consecutive days of treatment after OV from day 4 *versus* first- and fourth-day treatment following the laceration injury). Thus, we propose that *Mmp9* and *Mmp13* decreased expression may contribute to the altered muscle growth of RhoA mutants by modulating mainly SC fusion. Many studies were conducted to show the importance of Mmps on muscle growth. Fiber size is decreased in adult hindlimb muscles from *Mmp9* null mice (Mehan et al., 2011) and increased in muscles harboring a specific overexpression of *Mmp9* (Dahiya et al., 2011). In the context of hypertrophic growth, Peterson’s laboratory recently proposed that Mmp9 levels in muscle fibers should be finely regulated (through miRNA secreted by SCs among others) to have a correct ECM integrity and to facilitate the long-term hypertrophic response; too much or not enough Mmp9 being deleterious (Murach et al., 2020). In line, continuous unregulated high Mmp9 activity disrupted SC niche through ECM damages (Chenette et al., 2016). *Mmp13* null mice did not exhibit any histological or functional muscle deficits; however, muscle hypertrophy caused by increased IGF-1 was impaired (Smith et al., 2020). Mmp9 and 13 have also been shown to modulate myoblast migration *in vitro* (Lei et al., 2013; Lewis et al., 2000). It is reasonable to assume that the remodeling of ECM by Mmps enables more efficient movement of myoblasts toward myofibers and thus contributes to SC recruitment upon growth.

Among the genes mostly affected in RhoA Mut muscles upon OV, we focused our interest on two potent macrophage attractants: *Cx3cl1* and *Ccl3* whose expression is increased upon OV in Ctl and not in Mut. Data obtained in Ctl and Mut isolated myofibers and in myotubes treated with a Rho activator plus an Erk inhibitor further demonstrated that RhoA stimulated the expression of these chemokines in a myofiber cell-autonomous and in an Erk1/2-dependent manner. We do not know whether Cx3cl1 and Ccl3 influence SCs directly, but we can strongly relate their decreased expressions to a significant impairment of macrophages recruitment in RhoA mutants. Indeed, OV induced a transient accumulation of macrophages in Ctl *plantaris* muscles that was blunted in Mut. However, we cannot exclude that the impairment of macrophage recruitment in Mut might be due to the alteration of ECM (Figure 5) (Sapudom et al., 2020). In humans, it has been shown that resistance exercise increases the expression of chemotaxic factors including *Cx3cl1*, mobilizes monocytes (Della Gatta et al., 2014; Jajtner et al., 2018; Strömberg et al., 2016), and that macrophage number positively correlates with myofibers hypertrophy (Walton et al., 2019). Knowing the multifaceted roles of macrophages during muscle regeneration to support tissue recovery, in particular by promoting SC differentiation (Chazaud, 2020) and establishing a transient SC activating niche providing proliferation-inducing cues (Du et al., 2017; Ratnayake et al., 2021), we proposed that macrophages may as well participate in muscle hypertrophy by facilitating SC recruitment to myofibers. By depleting macrophages in Ctl mice, we showed that macrophages were required for muscle hypertrophy. Our results are in agreement with those of DiPasquale who showed that macrophages depletion also negatively affected muscle hypertrophic growth (DiPasquale et al., 2007). However, we provide additional data concerning the positive action of macrophages on SC number and fusion.

It is important to note that the absence of RhoA in the myofibers is sufficient to affect the behavior of RhoA expressing SCs, highlighting the importance of RhoA signaling in myofibers for the remodeling of SC microenvironment upon hypertrophy. How the SC niche is remodeled during hypertrophy process remains poorly examined. It was already proved that after increased load, myofibers secrete myokines like IL-4 and IL-6 that are able to influence SC proliferation and fusion (Guerci et al., 2012; Serrano et al., 2008; Horsley et al., 2003), and it has been recently shown that SC-derived extracellular vesicle may participate in the creation of an environment facilitating hypertrophic growth (Murach et al., 2021b). In addition, important transcriptional modifications of the expression of several genes encoding ECM components (such as *Collagens*, *Fn1*, and *Tnc-C*) have been reported shortly after OV, in this study (1wk) and Carson’s study (3 days after OV in rat) (Carson et al., 2002). By affecting the expression of *Mmp9* and *Mmp13*, RhoA might promote an important global ECM rearrangement and a modification of SC niche upon OV that could influence SC behavior, such as fusion. We show protein accumulation of several Mmp9/13 ECM protein substrates such as Col1, Tnc-C, and Fn1. Moreover, analysis of collagen fibrils structure in OV Mut muscles displayed a significant increase in fibrillar collagen tortuosity as compared to Ctl. These data suggest that the correct rearrangement of ECM/collagen is crucial to build an SC microenvironment appropriated for muscle growth and SC fusion. Importantly, SCs are sensitive to their extracellular matrix environment and respond differently to different collagen architecture (Hu et al., 2021). Interestingly, both Tnc-C and Fn1 have been shown to remodel and adapt SC niche (Tierney et al., 2016) and to regulate a feedback signal to promote symmetric division and replenish the SC pool (Bentzinger et al., 2013), respectively. In both cases, the proteins are produced by SCs that auto-regulate their own niche. Because in our instance SCs are “wild type”, we could speculate that this auto-regulation is not sufficient alone but should be associated with enzyme activity for ECM remodeling from the myofiber. Of note, as the macrophage number is decreased in RhoA Mut compared with Ctl muscles upon OV, on top of a myofibers intrinsic deficit, one part of the defective ECM reorganization occurring in Mut could be attributed to macrophages which also secrete Mmp9 in a context of muscle regeneration (Rahman et al., 2020). Moreover, we should consider the influence of ECM on other cells type beyond SCs and on other muscle tissue components (like microvessels), and its crucial role in maintaining and stabilizing muscle structure. Failure of ECM modifies myofibers mechanical properties making them more load-resistant and increasing their susceptibility to mechanical stress (Kritikaki et al., 2021) that may influence their.

In conclusion, we have described an important role for RhoA within myofibers in regulating muscle hypertrophic growth and SC accretion. We propose that it is achieved through a modification of SC microenvironment by a fine ECM remodeling and by inflammatory cells recruitment. It will be important in future works to define more comprehensively the molecular pathways and the specific cellular contributions involved in the control of muscle mass, in particular in response to increased mechanical load. This may be essential to identify and design treatments able to alleviate loss of muscle mass during disuse or aging.

### Limitations of the study

Although we have identified RhoA signaling as a key pathway in myofibers during overload-induced hypertrophy by building a permissive environment, future *in vivo* rescue studies will be required to demonstrate the causal role of ECM remodeling and macrophage recruitment in the altered SC fusion. Furthermore, the relative contribution of protein synthesis and of SC recruitment in the altered growth response of RhoA mutant myofibers will demand further investigations.

## STAR★Methods

### Key resources table

**Table:** 

REAGENT or RESOURCE	SOURCE	IDENTIFIER
**Antibodies**		

Mouse anti-Dystrophin	Novocastra	Cat# NCL-Dys2; RRID: AB_442081
Rat anti-F4/80	Invitrogen	Cat# MA1-91124; RRID: AB_2277854
Rabbit anti-phospho-S6 Ribosomal protein (Ser235/236)	Cell Signaling	Cat# 4858; RRID: AB_916156
Rabbit anti-Laminin	Sigma	Cat# L9393; RRID: AB_477163
Mouse anti-puromycin antibody	Merck Millipore	Cat# MABE343; RRID: AB_2566826
Mouse anti-RhoA	Santa Cruz	Cat# sc-418; RRID: AB_628218
Rabbit anti-P-Akt	Cell Signaling	Cat# 9271S; RRID: AB_329825
Rabbit anti-Col1	Abcam	Cat# ab34710; RRID: AB_731684
Rabbit anti-Fibronectin	Sigma	Cat# F3648; RRID: AB_476976
Rabbit anti-Erk2	Cell Signaling	Cat# 9102; RRID: AB_330744
Rabbit anti-P-Erk1/2	Cell Signaling	Cat# 9101; RRID: AB_331646
Rat anti-TenascinC	Invitrogen	Cat# MA1-26778; RRID: AB_2256026
Rouse anti-Gapdh	Cell Signaling	Cat# D16H11; RRID: AB_11129865
Mouse anti-Pax7 antibody	Santa Cruz	Cat# sc-81648; RRID: AB_2159836
Rat anti-laminin	Sigma	Cat# L0663; RRID: AB_477153

**Bacterial and virus strains**		

Ad-mCherry	Vector Biolabs	Cat# 1767
Ad-Cre-mCherry adenoviruses	Vector Biolabs	Cat# 1771
Recombinant Lenti-GFP	This paper	NA

**Chemicals, peptides, and recombinant proteins**		

Clophosome-A and control liposome anionic	ChemQuest	Cat# F70101C-A-2
Tamoxifen	MP Biomedicals	Cat# 156,738
EdU	Life Technologies	Cat# A10044
GM6001	Merck Millipore	Cat# CC1010
Erk inhibitor binimetenib (MEK162)	Selleckchem	Cat# S7007
Puromycin	Sigma	Cat# CP7255

**Critical commercial assays**		

EnzChek™ Gelatinase/Collagenase assay kit	Invitrogen/Molecular Probes	Cat# E12055
Click-iT® EdU Alexa Fluor® 647 kit	Life Technologies	Cat# C10340

**Deposited data**		

Raw and analyzed data	This paper	GEO: GSE166597 and GEO: GSE166598

**Experimental models: Organisms/strains**		

Mouse: HSA-Cre-ER^T2^:RhoAflox/flox	Lauriol et al., 2014; Schuler et al., 2005	N/A
Mouse: Pax7-nGFP:RhoAflox/flox	Lauriol et al., 2014	
Sambasivan et al., 2009	N/A	

**Oligonucleotides**		

See Table S1 for a list of oligonucleotides	Eurogentec	N/A

**Software and algorithms**		

Ingenuity (IPA) software	Qiagen	https://digitalinsights.qiagen.com/products-overview/discovery-insights-portfolio/analysis-and-visualization/qiagen-ipa/
Prism 6.0 software	GraphPad	https://www.graphpad.com/scientific-software/prism/
ImageJ	Schneider et al., 2012	https://imagej.nih.gov/ij/
MuscleJ plugin	Mayeuf-Louchart et al., 2018	https://github.com/ADanckaert/MuscleJ/blob/master/MuscleJ_1_0_2.ijm

**Oligonucleotides (RT-qPCR)**		

RhoA F	Eurogentec	5′-AACCTGTGTGTTTTCAGCACC-3′
RhoA R	Eurogentec	5′-ACCTCTGGGAACTGGTCCTT-3′
MuRF1 F	Eurogentec	5′-GAATAGCATCCAGATCAGCAG-3′
MuRF1 R	Eurogentec	5′-GAGAATGTGGCAGTGTTTGCA-3′
MAFbx F	Eurogentec	5′-TGTGGGTGTATCGGATGGAGA-3′
MAFbx R	Eurogentec	5′-CTGCATGATGTTCAGTTGTAAGC-3′
Mmp9 F	Eurogentec	5′-TCCTACTCTGCCTGCACCACTAAAG-3′
Mmp9 R	Eurogentec	5′-CTGTACCCTTGGTCTGGACAGAAAC-3′
Mmp13 F	Eurogentec	5′-AGTTGACAGGCTCCGAGAAA-3′
Mmp13 R	Eurogentec	5‘-CACATCAGGCACTCCACATC-3’
Adam8 F	Eurogentec	5′-GCAGGACCATTGCCTCTACC-3′
Adam8 R	Eurogentec	5′-TGGACCCAACTCGGAAAAAGC-3′
Ccl3 F	Eurogentec	5′-TTCTCTGTACCATGACACTCTGC-3′
Ccl3 R	Eurogentec	5′-CGTGGAATCTTCCGGCTGTAG-3′
Cx3Cl1 F	Eurogentec	5′-ACGAAATGCGAAATCATGTGC-3′
Cx3Cl1 R	Eurogentec	5′-CTGTGTCGTCTCCAGGACAA-3′
Col1α2 F	Eurogentec	5′-GTAACTTCGTGCCTAGCAACA-3′
Col1α2 R	Eurogentec	5′-CCTTTGTCAGAATACTGAGCAGC-3′
Tnc-C F	Eurogentec	5′-CACACACCGCATCAACATCC-3′
Tnc-C R	Eurogentec	5′-GACGACTTCTGCAGCTTGGA-3′
Fn F	Eurogentec	5′-GGCCACACCTACAACCAGTA-3′
Fn R	Eurogentec	5′-TCGTCTCTGTCAGCTTGCAC-3′
Hmbs F	Eurogentec	5′-TGCACGATCCTGAAACTCTG-3′
Hmbs R	Eurogentec	5′-TGCATGCTATCTGAGCCATC-3′

**Oligonucleotides (PCR)**		

Cre F	Eurogentec	5′-CCTGGAAAATGCTTCTGTCCG-3′
Cre R	Eurogentec	5′-CAGGGTGTTATAAGCAATCCC-3′
RhoA lox F	Eurogentec	5′-AGGGTTTCTCTGTACGGTAGTC-3′
RhoA lox R	Eurogentec	5′-GCAGCTAGTCTAACCCACTACA-3′

### Resource availability

#### Lead contact

Further information and requests for resources should be directed to and will be fulfilled by the lead contact, Dr Athanassia Sotiropoulos (athanassia.sotiropoulos@inserm.fr).

#### Material availability

This study did not generate nor use any new or unique reagents.

### Experimental model and subject details

#### Mouse protocols

*RhoA*^*flox/flox*^ mice are homozygous for RhoA floxed alleles harbouring LoxP sites flanking exon 3 of endogenous *RhoA* gene (Lauriol et al., 2014). HSA-*Cre-ER*^*T2*^ transgenic mice express Cre-ERT2 recombinase under the control of human skeletal actin promoter (Schuler et al., 2005). Tg :Pax7-nGFP transgenic mice express nuclear localized EGFP under the Pax7 promoter (Sambasivan et al., 2009).

To investigate the effect of myofiber-specific RhoA-deletion in adult muscle, the mouse strain following mice were generated: HSA-*Cre-ER*^*T2*^:*RhoA*^*flox/flox*^*.* In all experiments, 3-month-old HSA-*Cre-ER*^*T2*^:*RhoA*^*flox/flox*^ mice (males and females) were given five intraperitoneal (i.p.) tamoxifen (Tmx, 1 mg/day; MP Biomedicals) injections to induce *RhoA* deletion and were referred as mutant mice (Mut). *RhoA*^*flox/flox*^ mice injected with Tmx were used as control mice (Ctl).

No statistical difference in body weights were observed after Tmx in HSA-*Cre-ER*^*T2*^:*RhoA*^*flox/flox*^ and *RhoA*^*flox/flox*^ mice up to 2 months post Tmx injection.

Mice were genotyped by PCR using the following primers: Cre-F 5′-CCTGGAAAATGCTTCTGTCCG-3′; Cre-R 5′-CAGGGTGTTATAAGCAATCCC-3′; RhoAlox-F 5′-AGGGTTTCTCTGTACGGTAGTC-3′; RhoAlox-R 5′-GCAGCTAGTCTAACCCACTACA-3′.

Overload-induced hypertrophy (OV) of *plantaris* muscles of Ctl and Mut mice was induced through the incapacitation of *soleus* and *gastrocnemius* muscles by sectioning their tendon. This procedure was achieved in both legs. During the process of OV, Mut mice were injected with TMX at day 3, 5 and 7 post OV. At the indicated time (1 and 3wk post OV), *plantaris* muscles were dissected and subsequently processed for histological analyses. When indicated mice were administered intraperitoneally with 50 or 20 μg/g EdU (Life Technologies) or with 20μg/g GM6001 (Merck Millipore).

All animal experiments were conducted in accordance with the European guidelines for the care and use of laboratory animals and were approved by the institutional ethic committee and French Ministry of Research (number A751402).

### Method details

#### Macrophage depletion

Clodronate liposome and control liposome (Clophosome-A and control liposome anionic) were purchased from ChemQuest. Mice were injected with Clodronate- or control liposomes (0.15 mL administrated intraperitoneally i.p.) 1 day prior overload-induced hypertrophy. Additionally, liposomes (Clodronate- or control) were continued to be administrated day 1, 4, 6, 9, 12, 15 and 18 post-surgery (0.1 mL, i.p.). The muscles were harvested 7 days or 21 days post compensatory hypertrophy. No statistical difference in body weights were observed after Clodronate injections in Tmx-treated HSA-*Cre-ER*^*T2*^:*RhoA*^*flox/flox*^ and *RhoA*^*flox/flox*^ mice.

#### MMP activity

Fourteen μm thick cryostat sections of *Plantaris* were air dried at room temperature and pre-incubated 5min in dye-quenched (DQ) buffer with 0.01% Triton X-100 (buffer supplied by the manufacturer, Invitrogen/Molecular Probes) thrice. Sections were then overlaid with a fluorogenic substrate DQ gelatin (Invitrogen/Molecular Probes) diluted 1/100 in the DQ buffer and incubated for 2 hr at 37°C. Slides were then rinsed 10 min in PBS containing 0.01% Triton X-100 thrice. Cleavage of the substrate by gelatinases results in increase of fluorescence intensity by unblocking of quenched fluorescence. After washing, sections were used for immunohistochemical staining. The gelatinolytic activity (fluorescein-conjugated DQ-gelatin), dystrophin labeling (Alexa 546) and nuclei (DAPI) were acquired using an IXplore Spinning Disk microscope with a 60X objective (PLAN APOCHROME, NA 1.42), coupled with a sCMOS Orca flash 4.0 V3 camera (Hamamatsu).

#### SUnSET

The SUnSET, or SUrface SEnsing of Translation, technique allows estimation of global protein synthesis in tissue of living animals, specifically involves the use of an anti-puromycin antibody for the immunological detection of puromycin-labeled peptides. Briefly we injected mice with puromycin (Sigma) at 0.04 μg/g body mass via an i.p. injection 30min before samples harvesting. Western blot was then performed with a mouse anti-puromycin antibody (1/1000 Merck Millipore) and HRP-conjugated anti-mouse secondary antibody. Puromycin incorporation was measured by Fusion FX system (Vilber company) with Comassie blue staining as loading control.

#### Multiphoton imaging and characterization of ECM topography and architecture

*Plantaris* muscle was excised and placed in 2% paraformaldehyde (PFA) for 1hr at 4°C and then included in agarose low gelling 6%. Muscle was then sliced in thick sections (300μm) using a Leica VT1200 S vibratome (Leica Microsystems, Wetzlar, Germany). Leica SP5 multiphoton microscope (Leica Microsystems, Wetzlar, Germany) coupled with an Ultra II Chameleon Coherent TI:sapphire laser (Coherent, Saclay, France) was used to image collagen fibers by second harmonic generation microscopy. The laser beam was circularly polarized and a Leica Microsystems HCX IRAPO 25×/0.95 W objective was used. From z-stack images, the tortuosity was calculated using collagen fibrils Feret’s diameter divided by length (ImageJ software) of at least 50 single collagen fibrils per sample, as described in (Stearns-Reider et al., 2017).

#### Primary muscle cell culture and recombinant virus transduction

Primary cultures were derived from hindlimb muscles of *RhoA*^*flox/flox*^ *mice* harbouring Pax7*-nGFP* transgene that allowed the prospective selection by flow cytometry (Fluorescence Activated Cell Sorting or FACS) of satellite cells as described in (Randrianarison-Huetz et al., 2018). In standard conditions, myoblasts were grown in growth medium (DMEM/F12, 2% Ultroser G (PALL Life Sciences), 20% Fetal Calf Serum) on plastic dishes coated with 0.02% Gelatin. For differentiation, myoblasts were seeded in Matrigel-coated dishes and cultured in differentiation medium (DMEM/F12, 2% Horse Serum). When indicated cells were treated with Rho activator II CN03 (Cytoskeleton, 2 and 4 μg/mL) and/or Erk inhibitor Binimetenib (Selleckchem, 6 μM).

To induce in culture the excision of the floxed *RhoA* allele in myotubes, *RhoA*^*flox/flox*^ myoblasts were differentiated for 3 days and then transduced with Ad-mCherry or Ad-Cre-mCherry adenoviruses (100MOI, Vector Biolabs).

To generate a stable *RhoA*^*flox/flox*^ cell line expressing GFP, RhoA^flox*/flox*^ myoblasts were transduced twice with recombinant Lenti-GFP (100MOI, Vector Biolabs) and 8 μg/mL of polybrene then selected by FACS sorting.

#### Cell mixing fusion assays

To analyze fusion, myotubes (at day 2 of differentiation) were transduced with Ad-Cre-mCherry or Ad-mCherry. Two days post transduction, they were co-cultured with Lenti-GFP transduced myocytes (myoblasts incubated overnight in DM). Two days later, cultured cells were fixed for 8 min in 4% PFA and nuclei were stained using DAPI and then mounted in Dako Fluorescence Mounting Medium and kept at 4°C until image acquisition. Fusion events were scored by counting the dual-labeled cells (green cells GFP^+^/mCherry^+^). The number of fusion events was normalized by the total number of nuclei.

#### Single fiber isolation

*Plantaris* muscles were dissected from the legs by handling tendons only. Muscles were then placed in Collagenase Type I solution (2 mg/mL in DMEM; Life Technologies) and incubated in shaking water bath at 35°C for 50 min. Muscles were transferred to DMEM-filled horse serum–coated Petri dishes (to prevent fiber attachment to the plastic) using heat-polished glass Pasteur pipettes (with bore sizes that are just big enough to let the muscle go through). One at a time, muscles were triturated until fibers were separated. The bulk of fibers were then transferred to a Petri dish, and the dish containing the isolated fibers was placed in the culture incubator. The fibers should not be hypercontracted. Under the dissecting scope they should look long thin and shiny. After 15min, the damaged fibers shrink and the good fibers were collected, washed in PBS and freezed in liquid nitrogen in order to perform RNA extraction later.

#### Muscle section immunostaining

*Plantaris* muscles were collected and snap-frozen in liquid nitrogen-cooled isopentane. Eight μm-thick muscle sections were fixed in 4%PFA for 8 min at room temperature and blocked overnight at 4°C in PBS 1×, 10% Horse serum, 0.5% Triton X-100. Then, they were incubated with primary antibodies overnight at 4°C in PBS 1×, 10% Horse serum, 0.5% Triton X-100. The following primary antibodies were used: mouse anti-Dystrophin (Novocastra, NCL-Dys2, 1/50), anti-F4/80 (Invitrogen, MA1-91124, 1/400), rabbit anti-phospho-S6 Ribosomal Protein (Ser235/236) (Cell Signaling, 4858, 1/100), rabbit anti-Laminin (Sigma, L9393, 1/200) or rat anti-laminin (Sigma, L0663, 1/100). After washes in PBS 1x, sections were incubated with secondary antibodies for 1hr at room temperature. The following secondary antibodies used were goat anti-mouse IgG1 Alexa 488 (ThermoFisher, A21121, 1/1000) and donkey anti-rabbit Alexa 546 (LifeTechnologies, A10040, 1/1000). Nuclei staining were performed using DAPI. Muscles sections were then mounted in Dako Fluorescence Mounting Medium and kept at 4°C until image acquisition.

For Pax7 staining, muscle sections were fixed in 4%PFA for 8 min at RT and permeabilized in ice-cold methanol for 6 min. Muscle sections were treated with Antigen Unmaking Solution pH6 (Vector, H-3300) 15 min at 95°C and cooled on ice for 30 min. Blocking and incubation with primary and secondary antibodies were conducted as described in the previous paragraph. Primary mouse anti-Pax7 antibody (Santa Cruz, sc-81648) was used at dilution 1/50.

EdU detection was performed using Click-iT EdU Alexa Fluor 647 kit, according to the manufacturer’s instructions (Life Technologies).

#### Western blot analysis

*Plantaris* muscles were lysed in RIPA buffer (Sigma) and proteins were separated through denaturating SDS-PAGE electrophoresis using Mini-Protean TGX precast gels 4-15% (Biorad) and transferred on PVDF or Nitrocellulose (0.2μm, Biorad) membrane using the Trans-Blot turbo transfer system (Biorad). Membranes were blocked with 5% skinned milk in TBS-1% Tween (TBST) 1hr at room temperature and probed overnight at 4°C with primary antibodies in TBST 5% BSA. The following antibodies were used: mouse anti-RhoA (Santa Cruz, sc-418, 1/150), rabbit anti-P-Akt (Cell Signaling, #9271S, 1/1000), rabbit anti-Col1 (Abcam, ab34710, 1/1000), rabbit anti-Fibronectin (Sigma F3648, 1/1000), rabbit anti-Erk2 (Cell Signaling, #9102, 1/1000), rabbit anti-P-Erk1/2 (Cell Signaling, #9101, 1/1000), rat anti-TenascinC (Invitrogen, MA1-26778, 1/200), and mouse anti-Gapdh (Cell Signaling, D16H11, 1/1000). Following washing in TBST, membranes were hybridized with secondary antibodies goat anti-mouse- or goat anti-rabbit-coupled to HRP (ThermoFisher, 62–6520 and A27036, 1/10,000). Proteins were revealed using SuperSignal West Femto substrate (ThermoFisher).

#### Image acquisition

Fluorescence images were acquired using an Olympus BX63F microscope with 10× (UplanFL, NA 0.3×) and 20× objectives (UPLSAPO, NA 0.75), coupled with an ORCA-Flash4.0 LT camera (Hamamatsu), or using a Zeiss Axiovert 200M microscope with 5× (PLANFLUAR, NA 0.25×) and 20× objectives (LD PLANNEOFLUAR, NA 0.4), coupled with a CoolSnap-HQ camera (Photometrics), or using a Spinning Disk confocal microscope (Yokogawa X1) with a 100× oil-immersion objective (HCX PL APO, NA 1.47), coupled with a CoolSnap-HQ^2^ camera (Photometrics) and Metamorph 7.7.5 (Molecular Devices). Images were merged and edited in ImageJ. Background was reduced using brightness and contrast adjustments applied to the whole image.

#### Morphometric analysis

Myofiber cross-section area (CSA) was analyzed by using immunostaining of Dystrophin, marking myofiber sarcolemma, and then using MuscleJ tool (Mayeuf-Louchart et al., 2018) or ImageJ macro previously developed in our laboratory (Randrianarison-Huetz et al., 2018). Between 600 and 800 myofibers were analyzed. For the quantification of the number of nuclei per myofibers ImageJ was used and at least 500 myofibers were counted per muscle.

#### RNA extraction and RT-qPCR

Total RNA was extracted using TRIzol reagent and reverse-transcribed with SuperScript III reverse transcriptase (Invitrogen). cDNA was synthesized from 1μg of RNA. Quantitative PCR analysis was performed using a Light Cycler (Roche) according to the manufacturer’s instructions using a SYBR Green I kit (Roche). Values were normalized using *Hydroxymethylbilane synthetase* (*Hmbs*). The following primers were used: RhoA-F 5′-AACCTGTGTGTTTTCAGCACC-3′; RhoA-R 5′-ACCTCTGGGAACTGGTCCTT-3′; MuRF1-F 5′-GAATAGCATCCAGATCAGCAG-3′; MuRF1-R 5′-GAGAATGTGGCAGTGTTTGCA-3′; MAFbx-F 5′-TGTGGGTGTATCGGATGGAGA-3′; MAFbx-R 5′-CTGCATGATGTTCAGTTGTAAGC-3′; Mmp9-F 5′-TCCTACTCTGCCTGCACCACTAAAG-3′; Mmp9-R 5′-CTGTACCCTTGGTCTGGACAGAAAC-3′, Mmp13-F 5′-AGTTGACAGGCTCCGAGAAA-3′; Mmp13-R 5‘-CACATCAGGCACTCCACATC-3’; Adam8-F 5′-GCAGGACCATTGCCTCTACC-3′; Adam8-R 5′-TGGACCCAACTCGGAAAAAGC-3′; Ccl3-F 5′-TTCTCTGTACCATGACACTCTGC-3′. Ccl3-R 5′-CGTGGAATCTTCCGGCTGTAG-3′; Cx3Cl1-F 5′-ACGAAATGCGAAATCATGTGC-3′; Cx3Cl1-R 5′-CTGTGTCGTCTCCAGGACAA-3′; Col1α2-F 5′-GTAACTTCGTGCCTAGCAACA-3′; Col1α2-R 5′-CCTTTGTCAGAATACTGAGCAGC-3′; Tnc-C-F 5′-CACACACCGCATCAACATCC-3′; Tnc-C-R 5′-GACGACTTCTGCAGCTTGGA-3′; Fn-F 5′-GGCCACACCTACAACCAGTA-3′; Fn-R 5′-TCGTCTCTGTCAGCTTGCAC-3′; Hmbs-F 5′-TGCACGATCCTGAAACTCTG-3′; Hmbs-R 5′-TGCATGCTATCTGAGCCATC-3′.

#### Affymetrix microarrays

Microarray analysis was performed from: i) four *plantaris* muscles coming from four different mice per group. Four conditions (groups) were analyzed: SO Ctl, 1wOV Ctl, SO Mut, 1wOV Mut; ii) three independent *RhoA*^*flox/flox*^ differentiated myoblast cultures transduced with Ad-mCherry or Ad-Cre-mCherry adenoviruses for 2 days. Total RNAs were obtained using TRIzol reageant and DNAse treatment (Qiagen). RNA integrities were certified on bioanalyzer (Agilent). Hybridation to Mouse Gene 2.0-ST arrays (Affymetrix) and scans (GCS3000 7G expression Console software) were performed on the Genom’IC plateform (Institut Cochin, Paris). Probe data normalization and gene expression levels were processed using the Robust Multi-array Average (RMA) algorithm in expression Console software (Affymetrix). Gene ontology analysis was performed using Ingenuity (IPA) software.

### Quantification and statistical analysis

Quantitative data sets were analyzed using an unpaired parametric t-test with Welsh correction (Figures 1B, 1C, 4B, 6B, 6E, 7E, S4B, and S4D) (when two groups were analyzed), two-way ANOVA with Sidak’s multiple comparisons test (Figures 1D, 1F, 1G, 1H, 2A, 2B, 2C, 2E, 2H, 3D, 3E, 4A, 5A, 5B, 5C, 5D, 6C, 7A, 7C, 7E, 7F, 7G, S1A, S1B, S2A–S2D, andS3) (when two categorial variables were analyzed), one-way ANOVA with Sidak’s multiple comparisons test (Figures 4C, 6B, 6D, 6E, 6F, 6G, and 7D) (for three or more groups), using GraphPad Prism 6.0 software. Statistical significance was set at a p value<0.05.

## Data Availability

Transcriptomic data have been deposited in the Gene Expression Omnibus as accession GEO: GSE166597 and GEO: GSE166598. This paper does not report original code. Any additional information required to reanalyze the data reported in this paper is available from the lead contact upon request.
