# Supplementary material for: RhoA within myofibers controls satellite cell microenvironment to allow hypertrophic growth
Source: iScience. 2021 Dec 11;25(1):103616. doi: 10.1016/j.isci.2021.103616 (PMC8786647; doi:10.1016/j.isci.2021.103616)
Supplement: Document S1. Figures S1–S6 and Tables S1–S4 [file mmc1.pdf]

iScience, Volume *25*

Supplemental information

RhoA within myofibers controls satellite cell microenvironment to allow hypertrophic growth

Chiara Noviello, Kassandra Kobon, Léa Delivry, Thomas Guilbert, Florian Britto, Francis Julienne, Pascal Maire, Voahangy Randrianarison-Huetz, and Athanassia Sotiropoulos

Figure S1

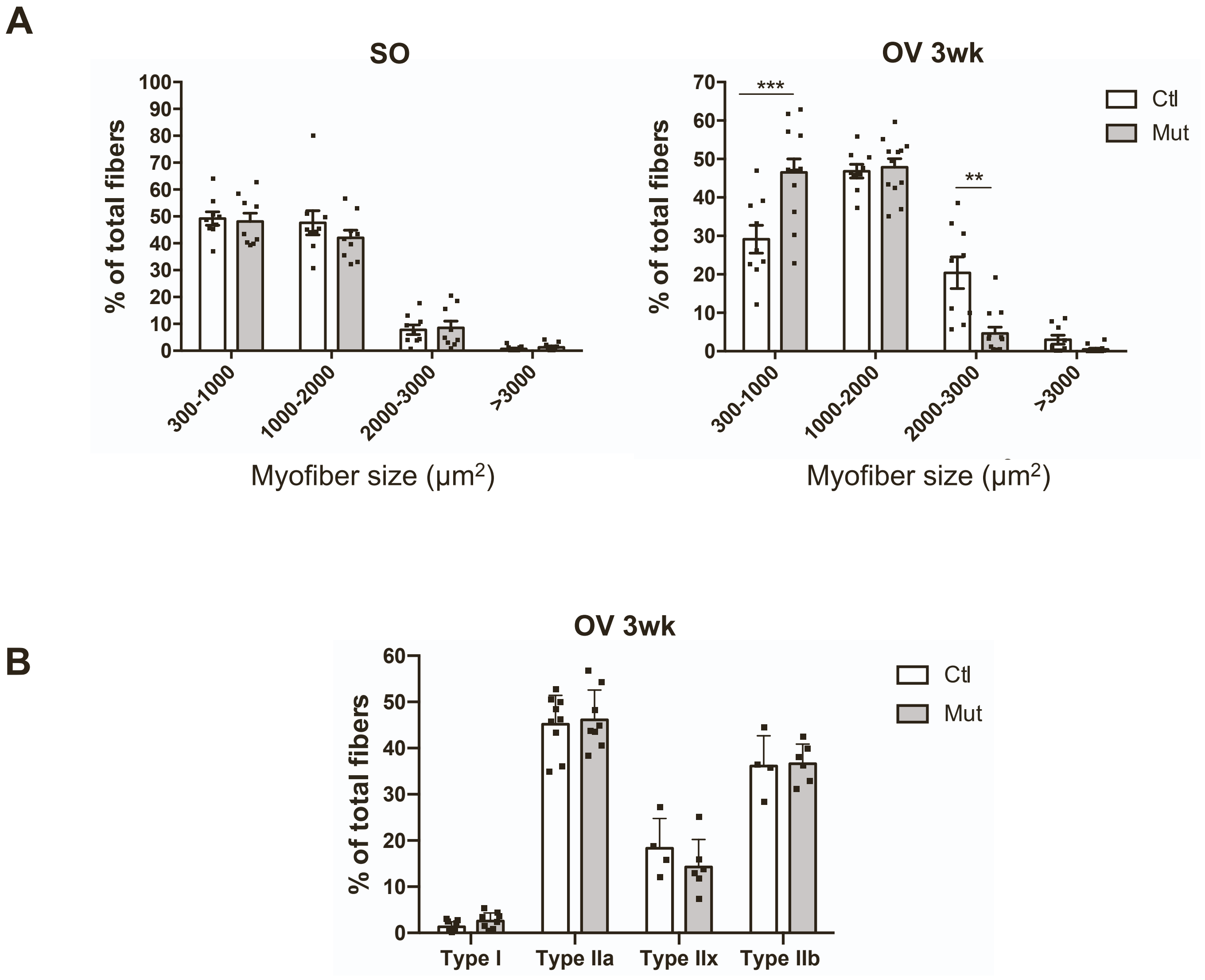

Figure S1. RhoA loss within myofibers does not affect myofiber type. **(A)** Myofiber CSA (µm^2) distribution of SO *Plantaris* muscle (left panel) and 3wk after OV (right panel) in Ctl and Mut mice (n=9-12). **(B)** Percentage of myofiber type in *plantaris* muscle after 3wk OV in Ctl and Mut (n=4-9). Data are mean±SEM. *pvalue<0.05, ***pvalue<0.001. Related to Figure 1.

Figure S2

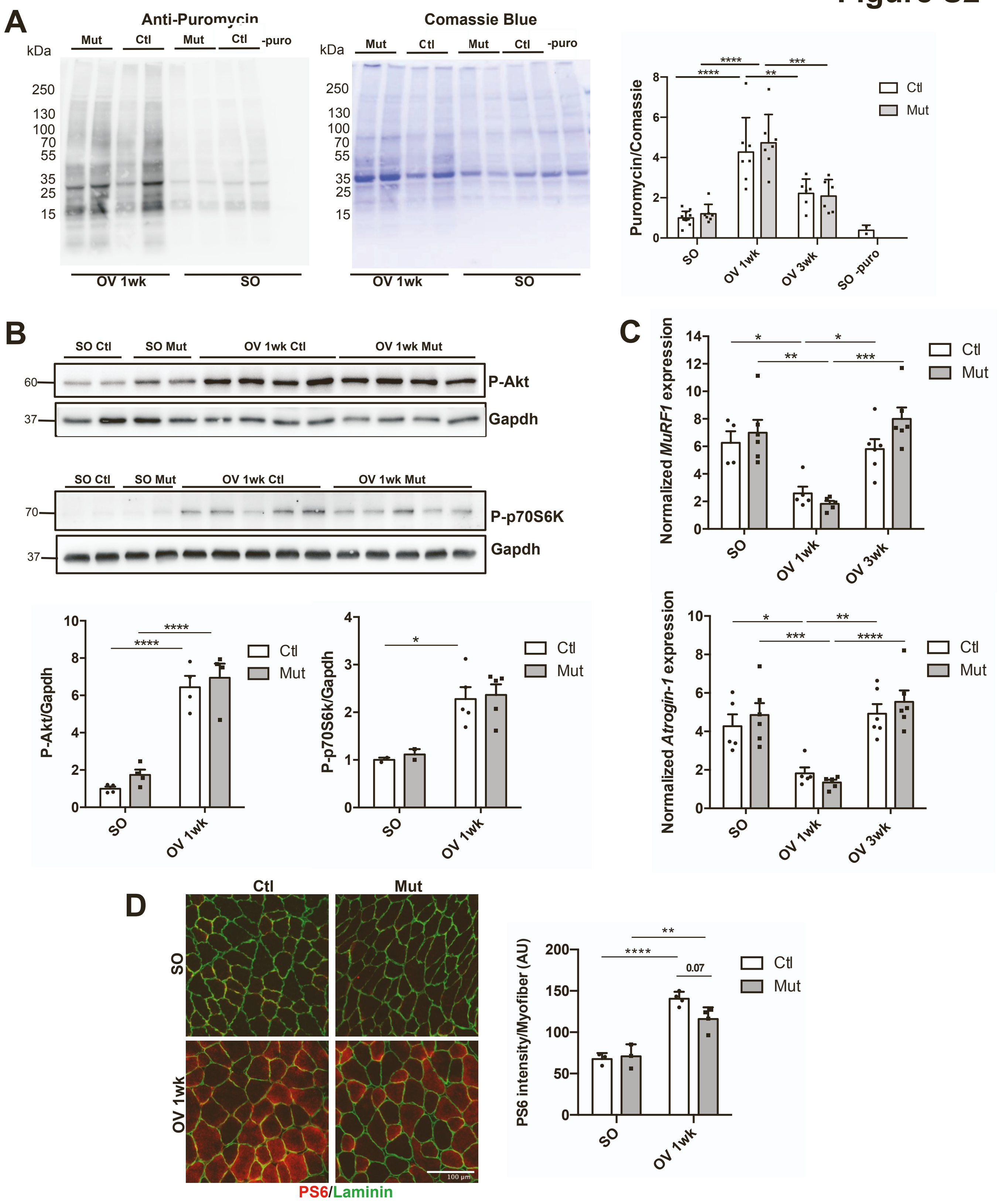

Figure S2. RhoA is not required for Akt dependent signaling and protein synthesis. (A) Representative SUnSET experiment performed on Ctl and Mut plantaris muscles before (SO) and after 1wk and 3wk OV. Puromycin was injected 30 min before harvesting. Puromycin-labeled peptides were quantified by Western blot using anti-Puromycin antibody. Coomassie was used as a loading control (n=4-8). Ratio of the quantification of Puromycin to Coomassie is shown in the right panel. **(B)** Phosphorylated Akt and p70S6K were analysed by Western blot in Ctl and Mut *plantaris* before (SO) and after 1wk OV. Gapdh was used as a loading control (n=4). Ratio of the quantification of P-Akt or P-p70S6K to Gapdh is shown in the right panel. **(C)** *MuRF1* and *Atrogin-1* mRNA expressions were analysed by RT-qPCR in Ctl and Mut *plantaris* before (SO) and after 1 and 3wk OV (n=4-6). Data were normalized by *Hmbs* expression. **(D)** Intensity of PS6 staining per myofiber on Ctl and Mut muscles sections before and after 1wk OV (n=3-4). Myofibers were delimitated using Laminin staining, PS6 fluorescence intensity was measured in 75 to 100 myofibers per condition. Data are mean±SEM. *pvalue<0.05, **pvalue<0.01, ***pvalue<0.001, ****pvalue<0.0001. Related to Figure 1.

Figure S3

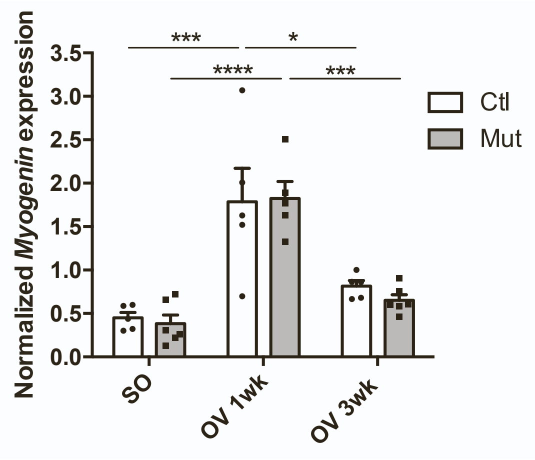

Figure S3. RhoA loss within myofibers does not affect *Myogenin* expression. *Myogenin* mRNA expression was analysed by RT-qPCR in Ctl and Mut *plantaris* muscles before (SO) and after 1 and 3wk OV (n=5-6). Data were normalized by *Hmbs* expression. Data are mean±SEM. **pvalue<0.01, ***pvalue<0.001, ****pvalue<0.0001. Related to Figure 2.

Figure S4

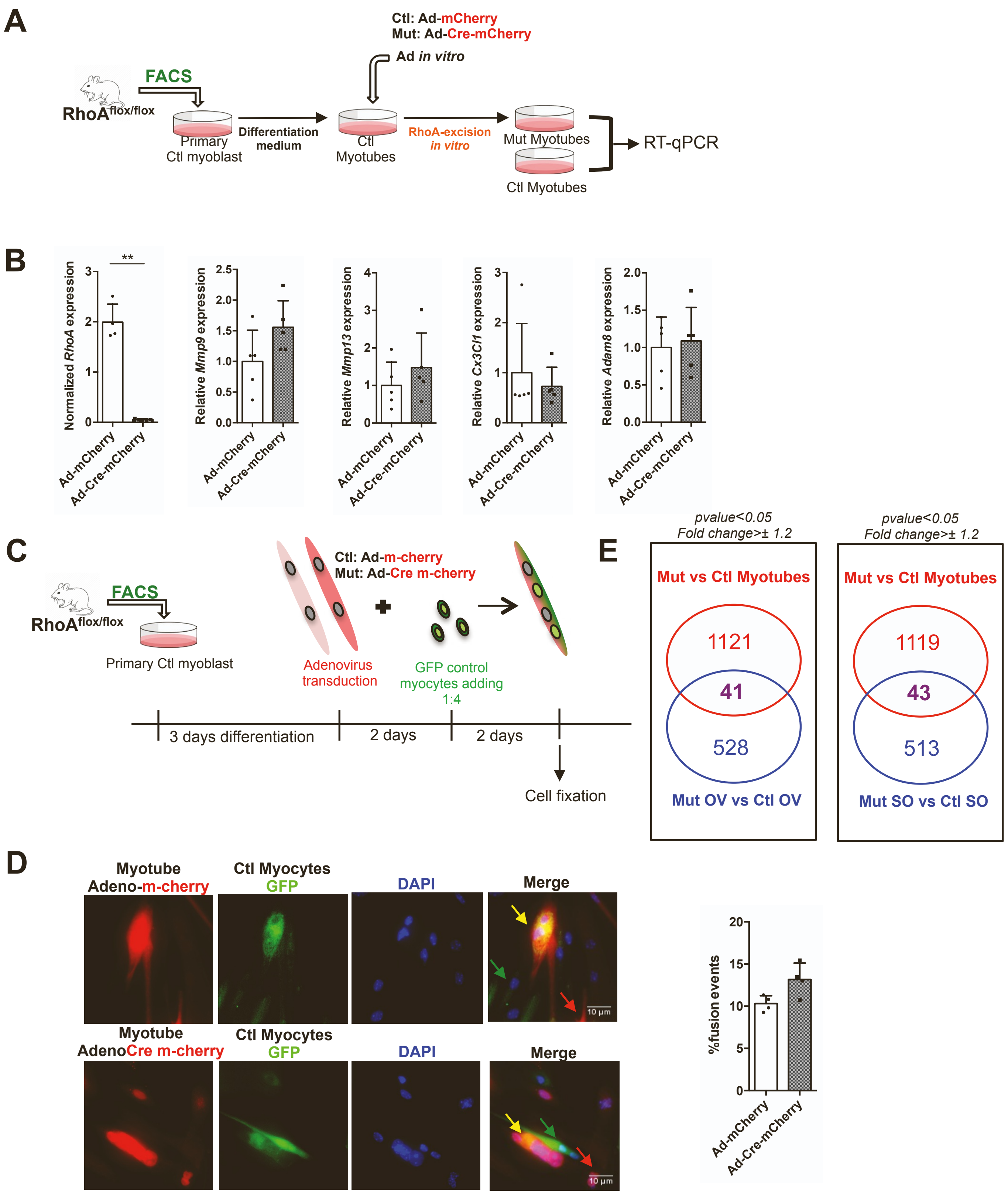

Figure S4. RhoA loss within myotubes *in vitro* does not affect myoblast fusion. (A) RhoA$^{flox/flox}$ Myotubes (at 3 days post differentiation) were transduced with Ad-mCherry (Ctl, red) or Ad-Cre-mCherry (Mut, red) and harvested 2 days later. **(B)** *RhoA, Mmp9*, *Mmp13*, *Adam8* and *Cx3Cl1* expressions were analysed by RT-qPCR *in RhoA$^{flox/flox}$* myoblasts transduced with Ad-mCherry or Ad-Cre-mCherry to induce RhoA loss. Data were normalized by *Hmbs* expression (n=4-5). **(C)** Myotubes transduced with Ad-mCherry (Ctl, red) or Ad-Cre-mCherry (Mut, red) were mixed with Ctl myocytes transduced with Lenti-GFP (green). After 48hr of co-culture, myotubes were analysed for dual labeling. **(D)** The percentage of dual-labeled cells per total number of nuclei (fusion events) was scored (n=3).**(E)** Affymetrix analysis has been performed from mRNA from Myotubes transduced with Ad-mCherry (Ctl) or Ad-Cre-mCherry (Mut). Venn diagram showing the intersection between genes differentially regulated by RhoA (fold change>±1.2; pvalue<0.05) in Myotubes, and *plantaris* overloaded muscles (Mut OV vs Ctl OV) or *plantaris* sham operated (Mut SO vs Ctl SO). Data are mean±SEM. ** pvalue<0.01. Related to Figure 3.

Figure S5

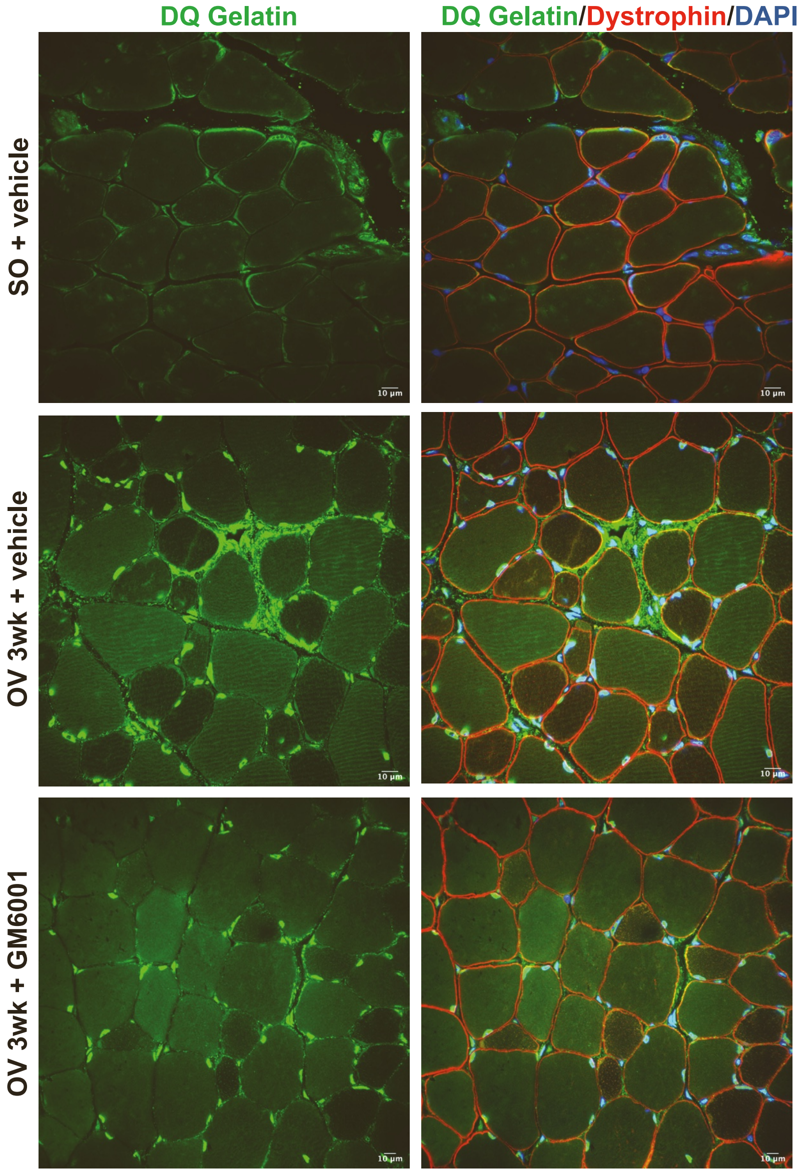

Figure S5. GM6001 treatment inhibits Mmps activity. Representative images of gelatinase activity (DQ-gelatin, green) in muscle section from vehicle- and GM6001-treated SO and OV mice. *In situ* zymography is represented in *Plantaris* muscle section. Note the OV induces prominent increase of the fluorescent signal both in the cell nuclei and in the intersitial space and that GM6001 treatment reduces gelatinase activity. Scale bar 10 µm. Related to Figure 7.

Figure S6

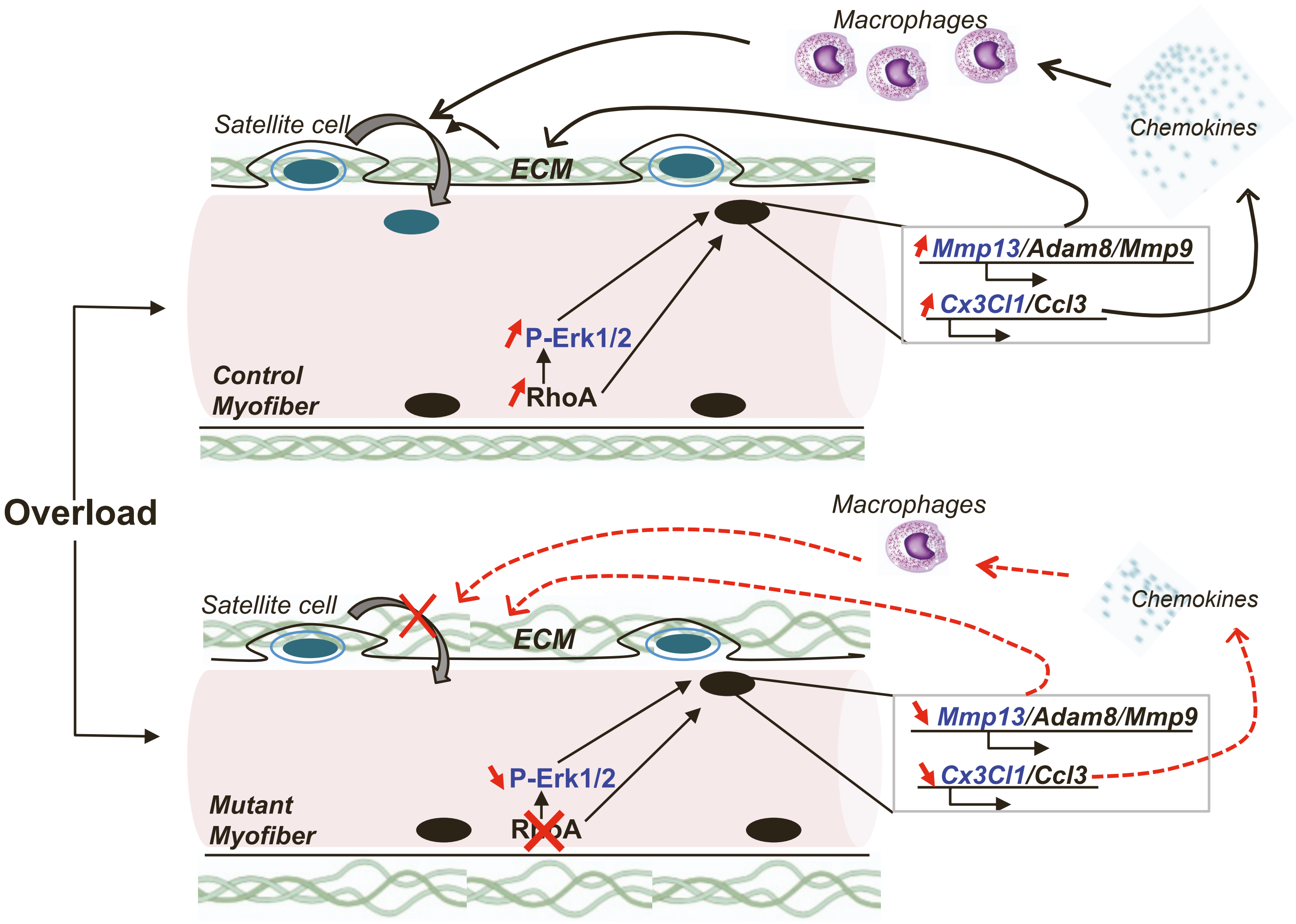

Figure S6. Schematic model: in response to increased workload, RhoA within myofibers gets activated and modulates *Mmp13/Cx3cl1* and *Mmp9/Adam8/Ccl3* expressions in a Erk-dependent and independent manner respectively. In turn, Mmp9/Mmp13/Adam8 modify ECM organization and Cxcl3/Ccl3 chemokines attract macrophages that will remodel SC microenvironment and thus support skeletal muscle hypertrophy and SC accretion. Related to Figure 1 to 7.

Table S1 - Microarray GO analysis in control *plantaris* 1 week after overload versus SO (related to Figure 1 and 3)

Canonical Pathways	pvalue	Z-score
Integrin Signaling	2.0E-11	4.07
IL-8 Signaling	5.6E-09	4.12
FcÎ³ Receptor-mediated Phagocytosis in Macrophages and Monocytes	6.6E-09	5.25
Remodeling of Epithelial Adherens Junctions	2.0E-08	2.23
Leukocyte Extravasation Signaling	5.6E-08	3.75
Regulation of Actin-based Motility by Rho	1.2E-07	4.23
**RhoA Signaling**	**2.5E-07**	**3.71**
Rac Signaling	4.7E-07	4.15
Glioma Signaling	8.1E-07	2.14
Phospholipase C Signaling	1.5E-06	3.16
Actin Nucleation by ARP-WASP Complex	1.5E-06	3.88
Cdc42 Signaling	2.0E-06	2.54
Dendritic Cell Maturation	4.3E-06	3.44
Colorectal Cancer Metastasis Signaling	6.3E-06	3.44
Signaling by Rho Family GTPases	6.8E-06	3.47
p70S6K Signaling	7.1E-06	2.56

Table S2 –Common genes differentially expressed in Myotubes expressing or not RhoA and in Control vs Mutant overloaded-*plantaris* that were similarly regulated (up or down) (related to Figure 3 and Figure S4)

	Myotubes lacking RhoA vs Ctl		Overloaded-plantaris Mut vs Ctl	
**Gene Symbol**	**Fold Change**	**pvalue**	**Fold Change**	**pvalue**
AmmerR1	-1.92	0.009	-1.233	0.00182
Bcl2A1	-1.63	0.0051	-1.623	0.0148
Csnk1g1	-1.76	0.004	-1.235	0.00366
Cst12	-1.33	0.0027	-1.221	0.00989
Lrp11	-1.91	0.0082	-1.25	0.022
RhoA	-1.68	0.0181	-1.204	0.00418
Slx4	-1.43	0.0054	-1.283	0.0117
Znf8	-1.44	0.0144	-1.216	0.00272
1700001F09Rik (includes others)	1.28	0.0481	1.306	0.0171
Akap1	2.78	0.0262	1.25	0.031
Arpc5l	1.46	0.0229	1.358	0.0146
Chchd1	1.35	0.0414	1.353	0.0218
Cog4	2.17	0.0284	1.267	0.0121
Cox19	1.69	0.0458	1.263	0.00886
Crygd	1.48	0.0129	1.369	0.021
Lonp1	1.5	0.044	1.246	0.0104
Mrpl12	1.58	0.0065	1.219	0.000396
Mrpl53	1.72	0.0404	1.248	0.0032
Ndufb5	1.23	0.0274	1.21	0.00386
Neurl2	1.81	0.0152	1.271	0.00937
Pipox	1.24	0.0384	1.267	0.0319
PolR2F	2.32	0.0366	1.302	0.00096
Ppa1	2.56	0.0391	1.226	0.0141
Timm10	1.86	0.0108	1.217	0.0458
Twf2	2.38	0.014	1.26	0.00321

Table S3 –Common genes differentially expressed in Myotubes expressing or not RhoA and in Control vs Mutant SO-*plantaris* that were similarly regulated (up or down) (related to Figure 3 and Figure S4)

	Myotubes lacking RhoA vs Ctl		Sham Operated-plantaris Mut vs Ctl	
**Gene Symbol**	**Fold Change**	**pvalue**	**Fold Change**	**pvalue**
Cep170	-2.93	0.0139	-1.254	0.0429
Cyth3	-2.12	0.0027	-1.274	0.0406
Eda2r	-1.96	0.0047	-1.34	0.0346
Sclt1	-1.74	0.0353	-1.235	0.0046
Arid2	-1.64	0.0181	-1.208	0.00991
Slc39a10	-1.63	0.0243	-1.221	0.0397
Hsd3b1	-1.62	0.0177	-1.364	0.0365
Tlr4	-1.52	0.0488	-1.375	0.0139
Optc	-1.48	0.0476	-1.259	0.0478
Ssbp2	-1.45	0.0322	-1.227	0.0147
C14orf28	-1.42	0.0421	-1.254	0.0213
Pus10	-1.42	0.0322	-1.298	0.0454
Gm10408 (includes others)	-1.4	0.0312	1.324	0.00291
Ptprc	-1.36	0.0341	-1.339	0.00811
Arl14epl	-1.35	0.0151	-1.273	0.0466
Mospd4	-1.29	0.044	-1.215	0.00784
1700009j07rik	-1.28	0.0091	-1.297	0.0159
Klk3	1.23	0.0449	1.26	0.000858
Hsd17b14	1.26	0.0196	1.212	0.0149
Klra7 (includes others)	1.28	0.0426	1.349	0.0155
Mrm3	1.43	0.0435	1.395	0.0333
Gm12253	1.46	0.0031	1.239	0.00801
Crygd	1.48	0.0129	1.4	0.0219
Tbc1d22a	1.51	0.0429	1.252	0.0156
Igfbp1	1.53	0.0111	1.254	0.019
Scgb2b27 (includes others)	1.53	0.028	1.22	0.0148
Polr2j	1.56	0.0448	1.391	0.00958
Cox19	1.69	0.0458	1.205	0.0387
Mrpl15	1.71	0.0283	1.261	0.013
Ifi35	1.72	0.0279	1.267	0.0303
Borcs8	1.8	0.0268	1.206	0.000825
Timm10	1.86	0.0108	1.336	0.0107
Dhrs11	2.39	0.023	1.247	0.0302
Tprn	2.75	0.025	1.372	0.0292

Table S4 – Predicted activated/inhibited Upstream Regulators in Control *plantaris* 1 week after overload vs SO but not in Mutant *plantaris* (related to Figure 3)

Function	Upstream Regulator		Activation Z-score	pvalue of overlap	Link with Mmps	Link with inflammation
**Modulation of ECM**	**Mmp9**	Peptidase	2.167	1.24E-04		
	**Postn**	Secreted extracellular matrix protein	2.199	4.17E-02		
	**TnC**	matricellular protein	2.427	1.73E-04	Mmp9 substrat Generation of integrin-activating peptides	
	**Cyr61**	matricellular protein	2.457	1.27E-05		
	**Loxl2**	enzyme	2.392	7.54E-03		
	**Plau**	peptidase/in ECM	2.825	1.02E-03		
**Integrin Receptor**	**Itga9**	transmembrane receptor	3.569	5.45E-03	Generation of integrin-activating peptides from TnC	
	**ItgaV**	transmembrane receptor	2.218	9.51E-04		
**Growth Factor**	**Pgf**	growth factor VEGF sub-family	2.929	1.45E-03		
	**Fgf**	group/growth factor	2.128	9.91E-03	Mmp9 substrat/Active	
	**Fgf7**	growth factor	2.643	6.68E-04	Mmp9 substrat/Active	
	**Gdf2**	growth factor	2.923	2.71E-02		
	**EgfR**	kinase/ growth factor receptor	2.721	2.24E-03		
**Cytokines**	**Faslg**	cytokine	2.583	3.56E-02	Cleaved by Mmm7	
	**Tnf (family)**	group/cytokine	3.455	9.01E-04	Mmp9 substrat	
	**TgfbR1**	cytokine receptor	2.308	3.35E-03	Generation of active Tgfb by Mmp9	
	**Il3**	cytokine	2.389	1.76E-03		Activation of macrophages
	**Il17Ra**	cytokine receptor	2.686	3.56E-02		
	**Gm-csfR**	group/cytokine receptor	3.229	8.76E-03		Macrophage survival
**Chemokine**	**Ccl2**	chemokine chemoattractant	3.630	4.59E-03		Recrutes monocytes
	**CcR1**	Ccl3 chemokine receptor	2.387	1.49E-06		Macrophage chemoattractant
	**CcR2**	CCL2 chemokine receptor	2.452	3.26E-03		Recrutes monocytes
	**CxCl1**	chemokine chemoattractant	2.800	1.62E-02	Mmp9 substrat /Active	Regulation of inflammatory response
	**CxCR4**	CXCL12/Sdf1 chemokine receptor	2.798	7.82E-03		
**Signaling**	**Mapk3/Erk1**	Kinase	2.790	1.19E-02		
	**Mapk8**	Kinase	2.344	6.34E-04		
	**Stat3**	transcription regulator	3.036	3.26E-07		
	**Rock2**	kinase	2.393	3.44E-03		
	**RelA**	transcription regulator Nfkb	2.408	2.18E-02		
	**Sn50 peptide**	Nfkb inhibitor	-2.608	2.11E-02		
**Other**	**SerpinA4**	protease inhibitor	-2.171	3.67E-03	Mmp9 substrat/Inactive	
